# Macrocyclization strategies for cyclic peptides and peptidomimetics†

**DOI:** 10.1039/d1md00083g

**Published:** 2021-06-29

**Authors:** Clément Bechtler, Christina Lamers

**Affiliations:** Department Pharmaceutical Sciences, University of Basel Klingelbergstr. 50 4056 Basel Switzerland christina.lamers@unibas.ch

## Abstract

Peptides are a growing therapeutic class due to their unique spatial characteristics that can target traditionally “undruggable” protein–protein interactions and surfaces. Despite their advantages, peptides must overcome several key shortcomings to be considered as drug leads, including their high conformational flexibility and susceptibility to proteolytic cleavage. As a general approach for overcoming these challenges, macrocyclization of a linear peptide can usually improve these characteristics. Their synthetic accessibility makes peptide macrocycles very attractive, though traditional synthetic methods for macrocyclization can be challenging for peptides, especially for head-to-tail cyclization. This review provides an updated summary of the available macrocyclization chemistries, such as traditional lactam formation, azide–alkyne cycloadditions, ring-closing metathesis as well as unconventional cyclization reactions, and it is structured according to the obtained functional groups. Keeping peptide chemistry and screening in mind, the focus is given to reactions applicable in solution, on solid supports, and compatible with contemporary screening methods.

## Abstract 2

Macrocyclization between head, tail or sidechains is a frequently employed strategy to enhance peptide and peptidomimetic stability, selectivity and affinity.

## Graphical abstract

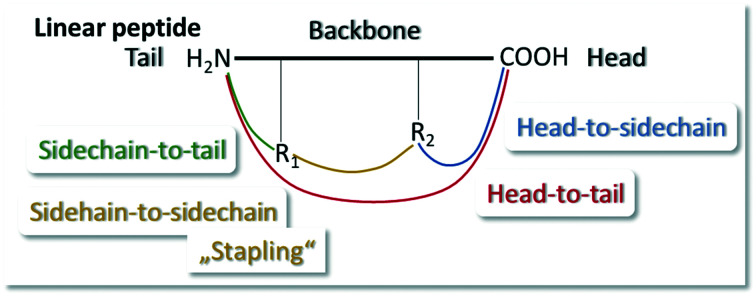

## 1. Introduction

Macrocyclic peptides are an interesting molecular format for drug discovery,^1^ combining the advantages of small-molecule and biological therapeutics: synthetic accessibility, low immunogenicity and toxicity, high binding affinity and selectivity, and the ability to target protein surfaces traditionally considered “undruggable”.^2–5^ Furthermore, macrocyclization renders peptides more stable and can increase membrane permeability,^6^ making it an important medicinal chemistry strategy in peptide drug development.^7^ Advances in high-throughput *in vitro* screening techniques have accelerated the identification of biologically potent macrocyclic peptides,^3,8,9^ and the field of macrocyclization is developing quickly to match.

The synthesis of cyclic peptides can be difficult to achieve by traditional methods, such as amide formation, because a defined pre-cyclization conformation must be formed, an entropically unfavorable process, before the desired intramolecular reaction can occur. This is especially true for head-to-tail cyclization, involving the cyclization of the C-terminus of the peptide with its N-terminus, because the preferred confirmation of amide bonds is all-*trans*, which leads to an extended peptide precursor.^10^ The introduction of turn-inducing elements is a strategy to circumvent this.^11,12^ Furthermore, most cyclizations need to be conducted in dilute solutions to favor the intramolecular reaction over intermolecular oligomerization.^12^ A pseudo-dilution effect can also be achieved by anchoring the peptide to an insoluble polymer, though this requires three-dimensional orthogonality in the protecting group strategy and anchoring *via* a sidechain when a head-to-tail cyclization is the goal.

Besides head-to-tail, peptides can also be cyclized head-to-sidechain, sidechain-to-tail or sidechain-to-sidechain. In particular, sidechain-to-sidechain cyclization has been extensively used to stabilize secondary structures, such as α-helices and β-sheets, yielding so-called ‘stapled petides’,^13,14^ or to generate protein epitopes^15,16^ and antibody CDR mimetics.^17^

In recent years, great progress has been made in identifying new cyclization strategies for peptide macrocyclization, spanning a wide range of chemistries from cross-coupling and photochemical reactions^18^ to enzymatic macrocyclization.^19^ Chemoselective reactions,^20^ reactions introducing orthogonality^21^ and diversity, are pushing the chemical space of macrocyclic peptides to new, more drug-like modalities. The chemoselective approaches in particular, such as ligations,^22,23^ will accelerate the increasing interest in peptide macrocycles since they allow peptide macrocyclization without the need for tedious protecting group strategies and are applicable to *in vitro* selection systems,^24^ accelerating lead identification.

In this review, we compiled the most important and modern organic chemistry macrocyclization strategies, structured by the produced connectivity. With this, we provided a concise overview for how to choose the appropriate reaction for peptide macrocyclization based on desired functional group. Finally, we summarized the different approaches in ESI† Table S1 to give the reader a short guide for selecting suitable reactions based on their specific requirements. To underline the importance of peptide macrocyclization in medicinal chemistry we highlight some applied examples and their bioactivities in ESI† Table S2.

## 2. Amide bond formation

### 2.1 Traditional amide cyclization

Many naturally occurring pharmacologically active peptides are cyclized head-to-tail, rendering them more resistant to hydrolysis by exopeptidases due to the absence of an N- and C-terminus. To cyclize a linear peptide precursor by amide bond formation, traditionally the same coupling chemistry is used as in linear peptide bond formation.^8^ However, conventional head-to-tail amide formation is non-trivial. Especially for head-to-tail cyclization of peptides shorter than seven residues, cyclodimerization and C-terminal epimerization can occur. In the retrosynthetic planning the ring disconnection must be chosen carefully, as for example, sterically hindered amino acids at the side of cyclization can reduce yields.^25^ To improve yields and reduce side product formation, preorganization of the peptide backbone can create a high effective molarity of the reaction partner. This can be done through turn-inducing elements such as proline, d-amino acids, or *N*-methylation.^12^ Conformational elements to pre-organize peptides for head-to-tail cyclization have been reviewed in detail.^12,26^

For amide formation, three main classes of peptide coupling reagents are used: carbodiimides, phosphonium reagents, and aminium−/uronium–iminium reagents.^27^ The careful choice of coupling reagent and additives can reduce epimerization.^9,28^ For example, PyBOP was used to complete the synthesis of cyclomarin C,^29^ whereas for teixobactin, a mixture of HATU/Oxyma Pure/HOAt/DIEA was preferred.^30^

Amide bond formation is not chemoselective, and in-solution cyclization requires sidechain-protected peptides, often rendering them poorly soluble. By forming the amide on solid support, the pseudo-dilution effect helps to reduce intermolecular reactions. In principle, two strategies have been used to achieve a head-to-tail cyclization on solid support: anchoring the peptide to the resin *via* the sidechain of a trifunctional amino acid^31^ or *via* the N-α atom of the C-terminus;^32^ the C-terminal carboxylate can react after orthogonal deprotection to form the cyclized product. Notable applications of head-to-tail and sidechain-to-sidechain lactam formation aim at the stabilization of secondary structures.^33^ For example, the design of a β-hairpin generated a protein–protein-interaction (PPI) inhibitor of the oncotarget p53-HDM2 that was smaller and had a higher activity compared to an α-helix (IC_50_ 0.53 μM *vs.* 1.1 μM, ESI† Table S2).^34^

### 2.2 Amide formation – sulfur mediated

In the last two decades, chemoselective reactions became the prevailing strategy for the synthesis of amide head-to-tail cyclized peptides.^22^ A multitude of different synthesis strategies employ *S*-to-*N* transfer in so-called ligation reactions, which have been recently reviewed.^35,36^

**Scheme 1:**
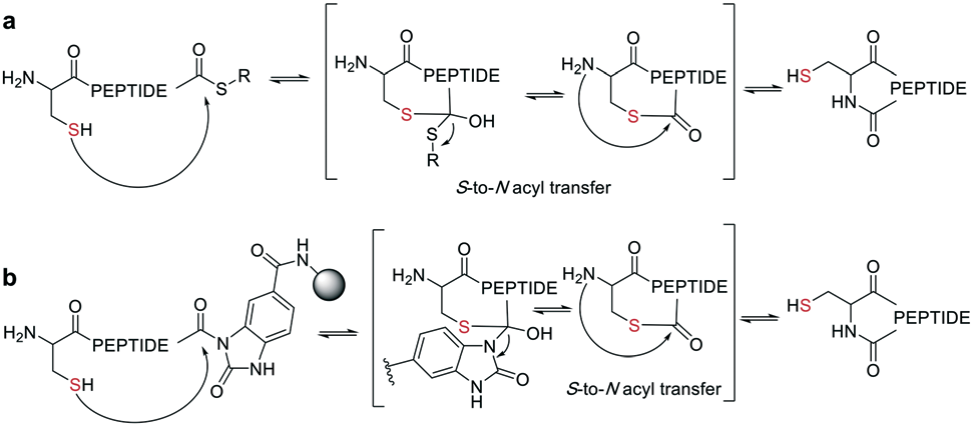
Reaction mechanism of sulfur mediated cyclization strategies: a: native chemical ligation. b: On solid support.

Native chemical ligation (NCL) was introduced as a mild and site-selective amide-formation reaction for synthesizing proteins from peptide fragments,^37^ and it was extended to the cyclization of peptides by Tam and coworkers.^38^ Here, an N-terminal cysteine reacts with a C-terminal thioester in neutral, aqueous solution. The reversibility of the transthioesterification step ensures chemoselectivity, since the irreversible *S*-to-*N* acyl transfer can only proceed at the N-terminal cysteine with its 1,2-aminothiol moiety (Scheme 1, a). This principle is exploited in the thia-zip peptide cyclization approach to access cyclotides.^39^ The reversible transthioesterification starts at the most C-terminal cysteine sidechain due to proximity to the thioester and proceeds in a sequential manner until it reaches the N-terminal cysteine, where the irreversible *S*-to-*N* acyl transfer occurs to stop the process. This is followed by a subsequent oxidation to form the intramolecular disulfide bond, which was reported to proceed smoothly in most examples.^39^

The excellent chemoselectivity of NCL can be explained by the poor nucleophilicity of other sidechains at pH 7. Thiols, such as PhSH or BnSH are added as nucleophilic catalysts to enable intermolecular transthioesterification. Low levels of epimerization and no oligomerization have been observed even at high concentrations.

On-resin NCL was first introduced by Muir *et al.* for Boc-SPPS on a buffer-compatible aminomethylated PEGA resin functionalized with thiol groups.^40^ Also, an Fmoc-SPPS-compatible NCL approach was reported, achieved by anchoring the sidechain of Asp to *p*-alkoxybenzyl ester as a linker for PEGA or CLEAR resin.^41^ A more recent strategy facilitating on-resin NCL uses a methyldiaminobenzoyl (MeDbz) linker to the resin, which is stable under Fmoc-SPPS conditions. After the assembly of the linear peptide sequence, MeDbz is then activated with 4-nitrophenyl chloroformate. Following global deprotection, the resin is treated with tris(2-carboxyethyl)phosphine (TCEP) in aqueous buffer (pH 6.8) to yield the cyclic peptide (Scheme 1, b).^42^

Though NCL is a powerful method for chemoselective head-to-tail peptide cyclization, there are limitations, such as the need for a cysteine in the peptide sequence. However, most of those have been tackled by desulfurisation,^43^ thiol-containing auxiliary groups, and cysteine surrogates, and the installation of the thioester has been accomplished *via* SPPS.^22^

**Scheme 2:**
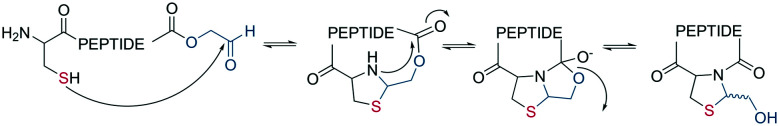
Reaction mechanism of cyclization generating a thiazolidine.

A different chemoselective reaction uses the 1,2-aminothiol of an N-terminal cysteine that readily condenses with an aldehyde to form a thiazolidine ring. By incorporating the aldehyde as an oxidized C-terminal glycolaldehyde ester, a head-to-tail cyclized peptide can be obtained by a ring-contraction mechanism proceeding *via* a tricyclic intramolecular rearrangement (Scheme 2).^44^

**Scheme 3:**
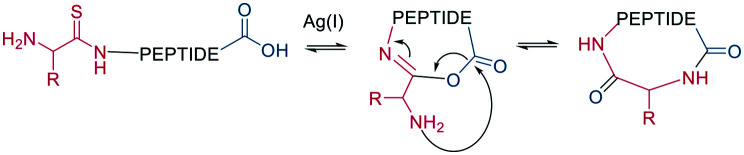
Head-to-tail peptide cyclization, AgI mediated.

As a contemporary approach, the head-to-tail ligation of a C-terminal carboxylic acid and a N-terminal thioamide can be promoted by AgI. Ag chemoselectively activates the N-terminal thioamide and brings it in proximity to the C-terminal carboxylate. An isoimide intermediate is formed after the extrusion of Ag_2_S and undergoes acyl transfer, resulting in a traceless macrocyclization. The thioamide is introduced as the last step of SPPS by coupling benzotriazole-based thioacylating reagents. Subsequently, the linear peptide is released from the solid support, and cyclization occurs *via* Ag_2_CO_3_ in DCM/MeCN (Scheme 3).^45^

**Scheme 4:**
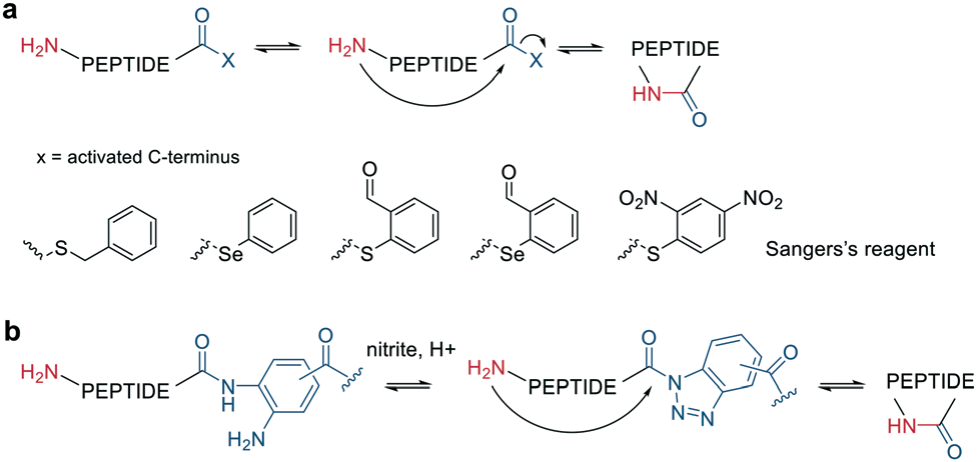
Reaction mechanism of aminolysis mediated cyclization strategy. a: In solution approaches with activated esters, b: on solid support.

Other chemoselective ligation reactions have been reported where the cyclization occurs by the attack of a nucleophilic amine at the mildly activated C-terminus. Houghten *et al.* reported an aminolysis of a C-terminal thioester in the presence of imidazole in an aqueous solution. However, this reaction is not chemoselective over the ε-amino group of Lys and shows epimerization.^46^ The use of other mildly activated esters (*e.g.*, selenoester,^47^ 2-formylthiophenol,^48^ selenobenzaldehyde^49^) can increase the reaction speed of the aminolysis, though their use has not yet been reported for peptide macrocyclizations (Scheme 4, a).^22^ Similarly, a C-terminal 9-fluorenylmethyl (Fm)-thioester reacts with the N-terminus when activated *in situ* by Sanger's reagent (1-fluoro-2,4-dinitrobenzene). The linear precursor can be synthesized in solution using Boc-chemistry except for the last amino acid, whose Fmoc protection will be removed simultaneously with the cleavage of the Fm-protected thioester to facilitate the reaction with Sanger's reagent and subsequent aminolysis (Scheme 4, a).^50^ By linking a linear peptide to a solid support using a diaminobenzyoyl (Dbz) linker, a macrocyclization by aminolysis can be achieved that is analogous to the NCL described above. The linear precursor is synthesized by Fmoc-SPPS, and the Dbz linker is subsequently activated by nitrite-mediated acyl benzotriazole formation to generate an activated C-terminus. This macrocyclization can be achieved under mild acidic conditions with the addition of HOAt and HOBt and in moderate to good yields (Scheme 4, b).^51^ The advantage of these aminolysis strategies over NCL is their applicability to any peptide sequence without the need for a specific amino acid, such as cysteine.

**Scheme 5:**
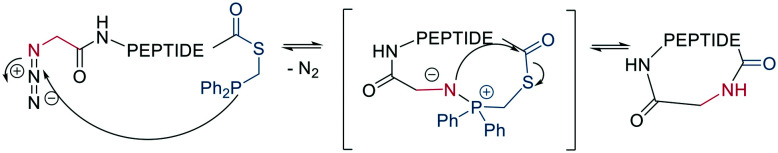
Peptide macrocyclization by Staudinger reaction.

Finally, a traceless Staudinger ligation can be used to head-to-tail cyclize a peptide in a chemoselective fashion. The C-terminal phosphino-thioester reacts with an N-terminal azide introduced by the noncanonical azidoglycine to yield a cyclic iminophosphorane, which collapses to an amide bond by eliminating the thiophosphorane (Scheme 5).^52^

### 2.3 Amide formation – mediated by other functional groups

**Scheme 6:**
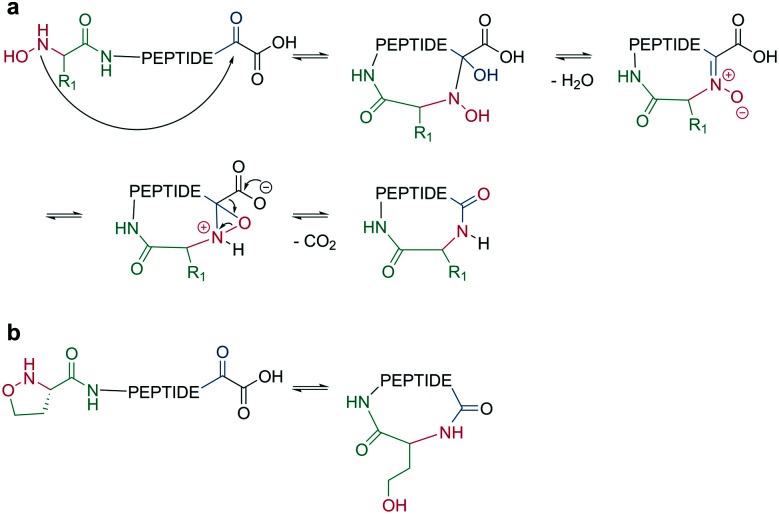
Cyclization by KAHA ligation. a: KAHA I with free hydroxylamine, b: KAHA II with 5-oxaproline.

Bode *et al.* reported a ligation reaction between a C-terminal ketoacid and the N-terminal hydroxylamine of proteins and peptides, termed the ketoacid-hydroxylamine (KAHA) ligation.^53,54^ This ligation yields macrocyclic peptides from unprotected linear peptides under mild conditions and in polar protic and aprotic solvents.^55^*O*-Substituted and cyclic hydroxylamines have been investigated to prevent oxidation of the N-terminal hydroxylamine. For example, 5-oxaproline was especially suitable for peptide synthesis, as it generated a homoserine depsipeptide that rearranged to a homoserine peptide by an *O*-to-*N* acyl shift.^56^ (Scheme 6, a and b). However, drawbacks of the KAHA ligation include a slow reaction, high epimerization rates, and the instability of hydroxylamines.

**Scheme 7:**
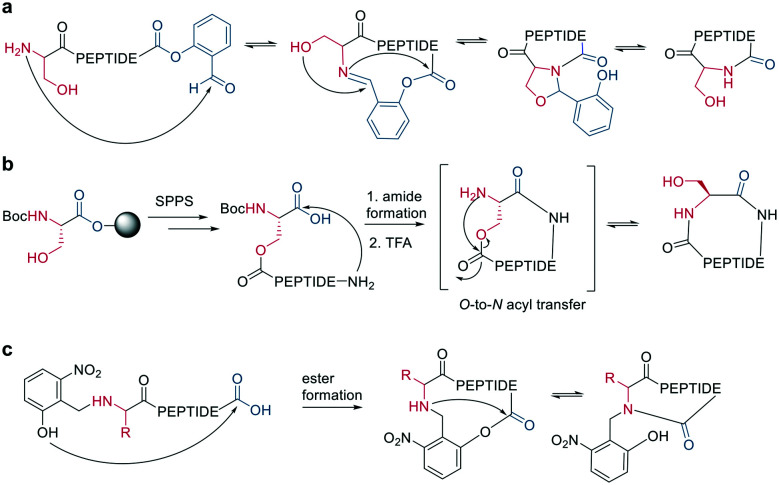
Reaction mechanism of: a: Ser/Thr ligation in solution, b: on solid support, c: cyclization with the help of auxiliary groups.

Ser/Thr ligation has been developed for the synthesis of proteins through the ligation of peptide fragments containing a serine or threonine.^57^ The C-terminal ester is activated as a salicylaldehyde ester, which is generated by the on-resin phenolysis of an *N*-acyl-benzimidazolinone (Nbz) linker with salicylaldehyde dimethyl acetal in Na_2_CO_3_, DCM/THF. This produces macrocycles without C-terminal epimerization.^57^ Ser/Thr ligation technology was extended to the backbone cyclization of tetrapeptides containing an N-terminal serine or threonine and C-terminal salicylaldehyde ester. The ligation of the unprotected peptide occurred in pyridine/acetic acid (1 : 2). After acidolysis with TFA/H_2_O, the cyclic peptides were obtained with no epimerization (Scheme 7, a).^58^

Like the *S*-to-*N* migration used in NCL, macrocyclization of a depsipeptide can be achieved by an *O*-to-*N* migration. An *N*-Boc-protected serine is coupled to a solid support, and the alcohol group is reacted with the subsequent Fmoc-amino acid to create an *O*-acyl isopeptide bond. The remaining amino acids are coupled using standard Fmoc-SPPS to generate the depsipeptide. After cleavage from the resin, the depsipeptide is cyclized by amide bond formation of the N-terminus and C-terminal carboxylic acid. Following the removal of the *N*-Boc group of the serine residue, the final *O*-to-*N* acyl migration takes place under basic conditions (Scheme 7, b). This strategy still relies on a conventional head-to-tail cyclization, though the depsipeptide strategy does enable the synthesis of penta- or hexapeptides, which are usually hard to cyclize. However, for a constrained tetrapeptide, this strategy was not successful.^59^

The usage of auxiliary groups is another strategy for cyclizing difficult sequences. For example, 2-hydroxy-6-nitrobenzaldehyde can be reacted with the N-terminus of the peptide, and an ester is formed by the attack of the phenol on the C-terminus. The following *O*-to-*N* acyl migration generates the lactam, and the auxiliary group can be released by exploiting its photo lability (Scheme 7, c).^60^

**Scheme 8:**
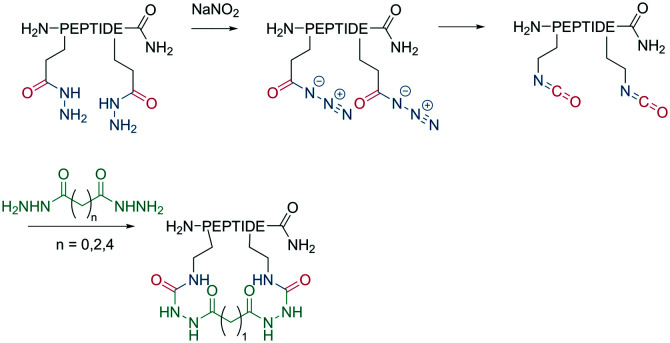
Reaction of isocyanates with dicarboxylic acid hydrazides on an unprotected peptide.

Analogous to amide bond formations, isocyanates generated *in situ* react with hydrazides to yield semicarbazides (Scheme 8). The reaction has good yields (60–77%) for different scaffold sizes (*e.g.*, *i*, *i* + 4; *i*, *i* + 7) and is a more robust cyclization for smaller ring sizes.^61^

### 2.4 Macrocyclization chemistry employing imines and oximes

**Scheme 9:**
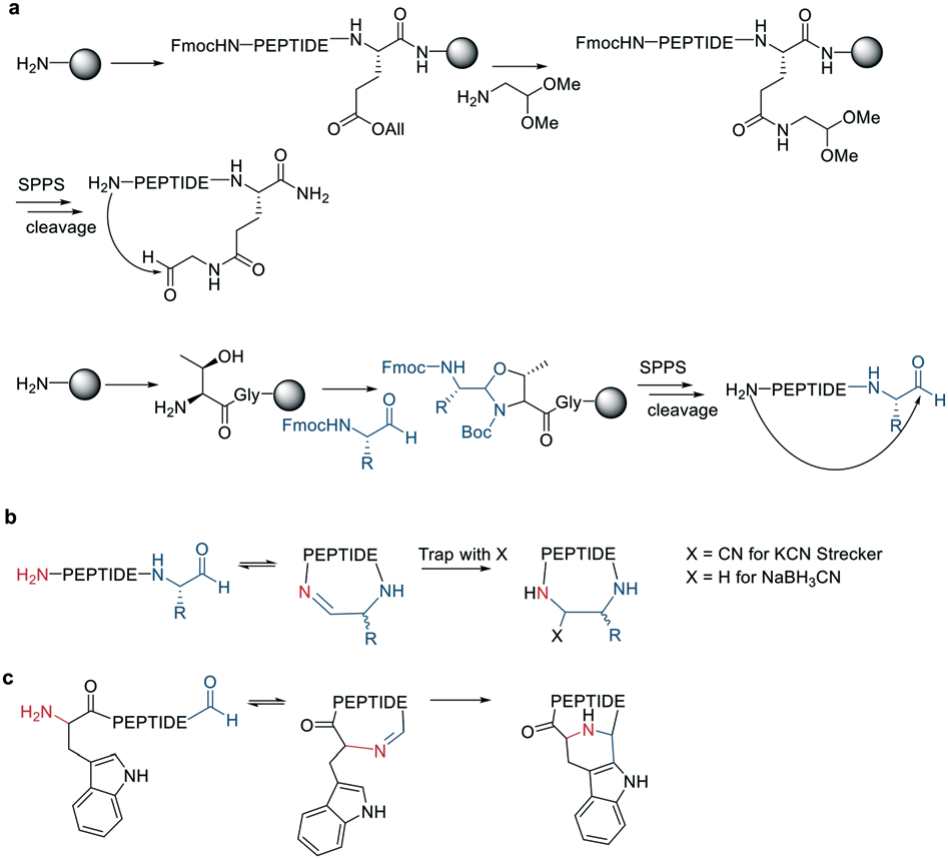
Peptide cyclization *via* imine formation and subsequently trapping of the imine. a: On-resin strategies to generate the aldehyde. b: General reaction scheme of imine formation and trapping. c: Trapping *via* Pictet–Spengler.

Inspired by natural products, Malins *et al.* developed new macrocyclization chemistry by forming an imine between an aldehyde and the N-terminal primary amine.^62^ To install the aldehyde, they applied two different, previously reported solid-phase approaches, either (i) installing an aldehyde on an aspartate sidechain by reacting it with amino acetaldehyde dimethyl acetal as a masked aldehyde unit, or (ii) coupling an α-amino aldehyde on a tyrosine-glycine resin. In the latter strategy, the C-terminal aldehyde becomes accessible upon cleavage from the resin (Scheme 9, a).^62^ Some of the tested peptide sequences readily cyclized in aqueous buffer after cleavage, while others remained linear, potentially due to the reversibility of imine formation. Therefore, different strategies to trap the imine have been reported, such as through the addition of nucleophiles like cyanide to trap the imine as α-aminonitriles. The resulting Strecker reaction proceeded in aqueous solution at room temperature in good yields while tolerating a broad range of sidechain functionalities, including Asp, Glu, Lys, His, Tyr, and Cys.^62^

Imines were also successfully and chemoselectively trapped as amines using NaBH_3_CN by reductive amination in aqueous NaOAc buffer (Scheme 9, b). Other intramolecular imine traps have been tested with aromatic rings, including indoles and imidazoles, which proceed *via* Pictet-Spengler macrocyclization (Scheme 9, c), and thio- or seleno-nucleophils, which trap the imine in a corresponding thia-/selenazolidine.^62^ The reaction is also selective for the N-terminal primary amine over the ε-amino group of Lys, which is proposed to be due to the difference in p*K*_a_.

**Scheme 10:**
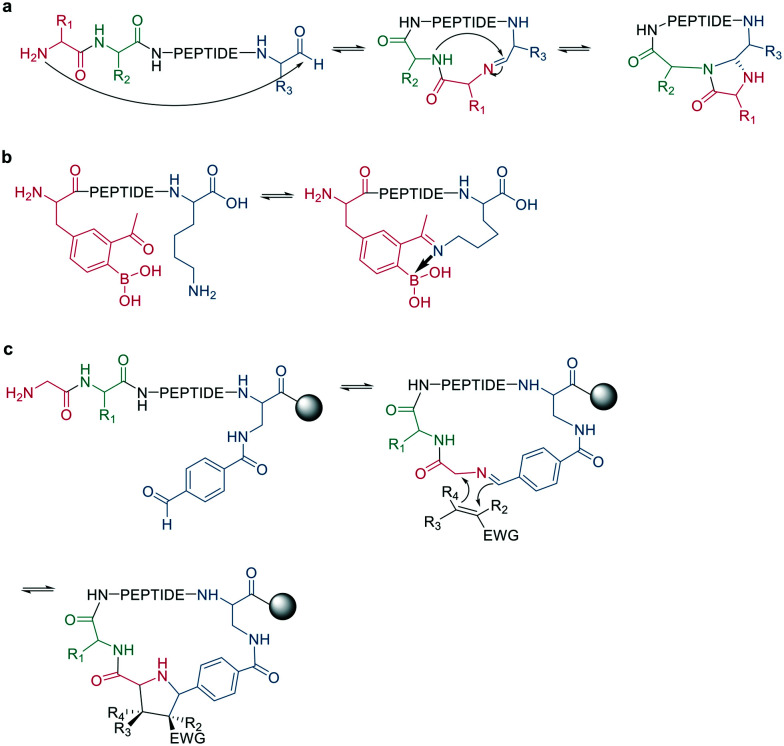
Peptide cyclization by a: 4-imidazolidinone, b: iminoboronate formation or c: dipolar cycloaddition.

Following imine formation, a nearby nitrogen can attack to generate a stable 4-imidazolidinone. Due to the high chemoselectivity for the intramolecular reaction, it can be carried out at high concentrations without an increase in oligomerization. Furthermore, the 4-imidazolidinone can act as a turn-inducing element, increasing intramolecular hydrogen bonds, conformational rigidity, and enzymatic stability. The reaction proceeds with high stereoselectivity, a high substrate scope, and fast kinetics in DMF/H_2_O (Scheme 10, a).^63^

Another strategy to trap an imine is by a boronic acid, which can be introduced in the peptide to allow the cyclization to proceed rapidly and spontaneously under physiologic conditions. Interestingly, this cyclization can be rapidly reversed in response to acids, oxidation, and α-nucleophiles (hydrazine and amino alcohols). At neutral pH (6.8), the macrocycle is stable, while acidic conditions hydrolyze the iminoboronate (Scheme 10, b). When reduced (NaCNBH_3_), the iminoboronate can be trapped irreversibly as aminoboronate in two diastereomers.^64^

A recent strategy traps the imine in a 1,3-dipolar cycloaddition, generating fused and spiro-ring systems that are frequently found in pharmacologically active natural products. The imine is generated on solid support by reacting 4-carboxybenzaldehyde with the primary amine of an N-terminal glycine (Scheme 10, c).^65^

**Scheme 11:**
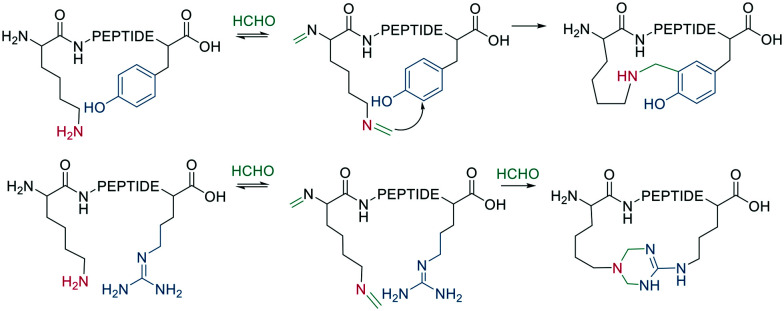
Cooperative macrocyclization of iminium with nearby arginine or tyrosine sidechain.

Native peptides reacted with formaldehyde to form an imine with the ε-amino group of Lys, which can crosslink to nearby tyrosine or arginine residues. Tyrosine reacts in the *ortho* position *via* a C-nucleophilic attack of the iminium ion intermediate and a subsequent re-aromatization (Scheme 11).^66^

**Scheme 12:**
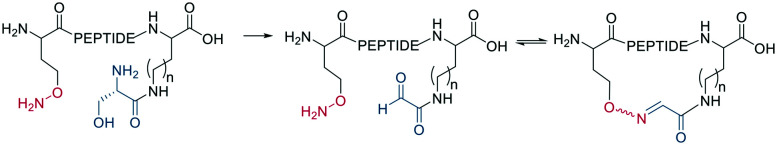
Macrocyclization by oxime formation.

Schiff bases (imines, hydrazones, and oximes) are used in dynamic covalent chemistry approaches due to their hydrolytic reversibility, with oximes being generally the most stable. Side-chain cyclization *via* oxime formation is achieved using noncanonical amino acids containing a 1,2-aminoalcohol, which is oxidized by NaIO_4_ to an aldehyde (Scheme 12). The aldehyde reacts with an noncanonical amino acid containing an aminooxy-sidechain to form an oxime in phosphate buffer (pH 7).^67^ Oxime formation is thermodynamically favored but kinetically slow at neutral pH. It can be accelerated by acidic conditions or nucleophilic catalysts. Importantly, oxime formation generates two isomers (*E* and *Z* oximes). When using aminooxy noncanonical amino acids, ethanedithiol should be added as a nucleophilic scavenger during cleavage to prevent the peptide from reattaching to the resin. Furthermore, aminooxy amino acids have been reported to bind irreversibly to the stationary phase of some C_18_ columns.^67^ Oxime formation has also been used to stabilize α-helical conformations (*i*, *i* + 4 spacing),^68^ and oxime chemistry can be applied for stapling peptides using noncanonical amino acids with amino-alcohol or hydrazine sidechains. By adding commercially available di-aldehyde scaffolds, the cyclization proceeds in phosphate buffer at pH 7.^69^

**Scheme 13:**
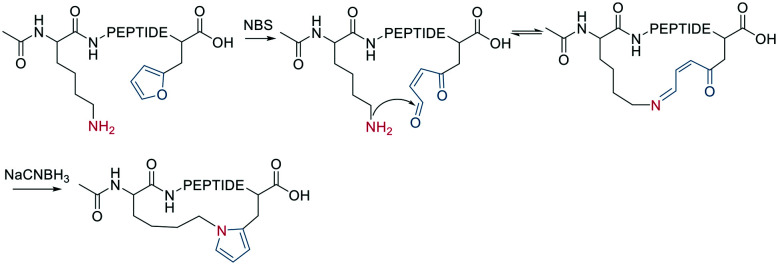
Oxidation of furan ring and subsequent cyclization with amino sidechain of lysine.

The noncanonical amino acid furanylalanine can be oxidized by *N*-bromosuccinimide (NBS) to form a ketoenal, which can react with nucleophilic sidechains (*e.g.*, Lys) to cyclize (Scheme 13). The reaction is irreversible after imine trapping by reduction with NaCNBH_3_ and can be applied as a one-pot reaction, though cysteine and tyrosine were not tolerated.^70^

### 2.5 Amine-reactive stapling

**Fig. 1:**
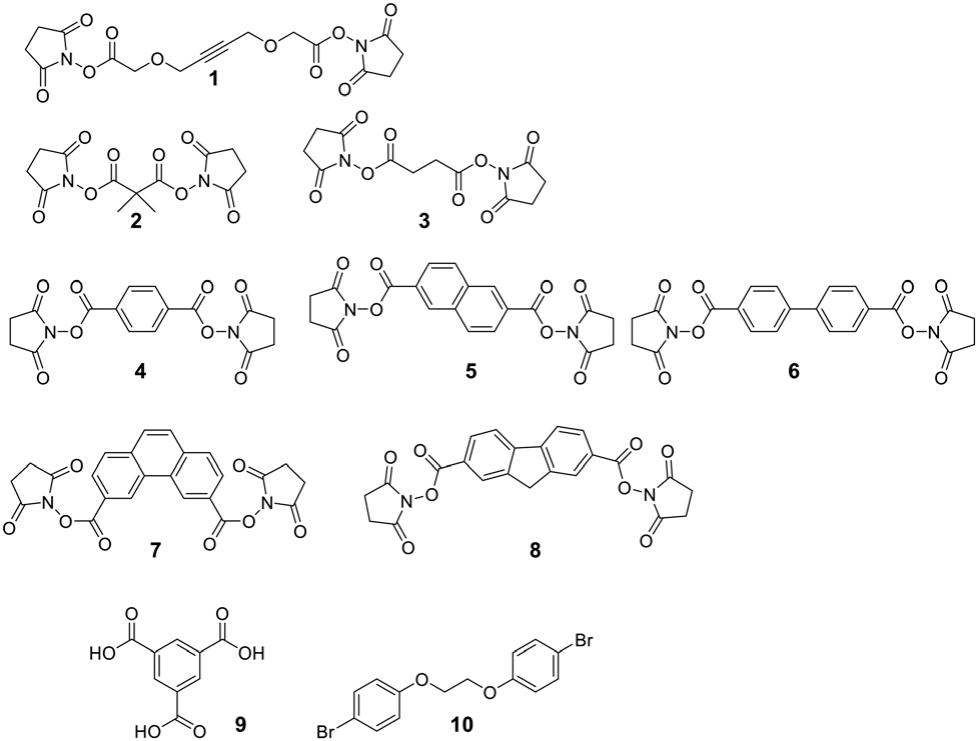
Examples of scaffolds used for amino-to-amino sidechain stapling.

A diverse set of amide-generating scaffolds for reactions with amines has been reported (Fig. 1). To cyclize peptides of an mRNA display library, a bifunctional NHS-scaffold (*e.g.*, disuccinimidyl glutarate) was used.^71^ Di-NHS scaffolds (**1–8**) generate crosslinked peptides stabilizing α-helical structures with different residue spacings (*i*, *i* + 4 ≈ 5 Å, *i*, *i* + 7 ≈ 11 Å, and *i*, *i* + 11 ≈ 16 Å).^72^ Using benzene-1,3,5-tricarboxylic acid (**9**) as an organic, planar, and tri-reactive scaffold, a bicyclic one-bead-two-compounds library was generated by amide formation to sidechain amino groups using PyBOP/HOBT/DIPEA. The library was applied to identify TNFα inhibitors with nanomolar affinities (450 nM), and the scaffold was essential to that affinity (linear sequence >10 μM, ESI† Table S2).^73^ Bicyclic scaffolds can also be used to target intracellular targets (*e.g.*, tyrosine phosphatase 1B), by encompassing a cell-permeable sequence in one cycle of the bicyclic peptides.^74^

**Fig. 2:**
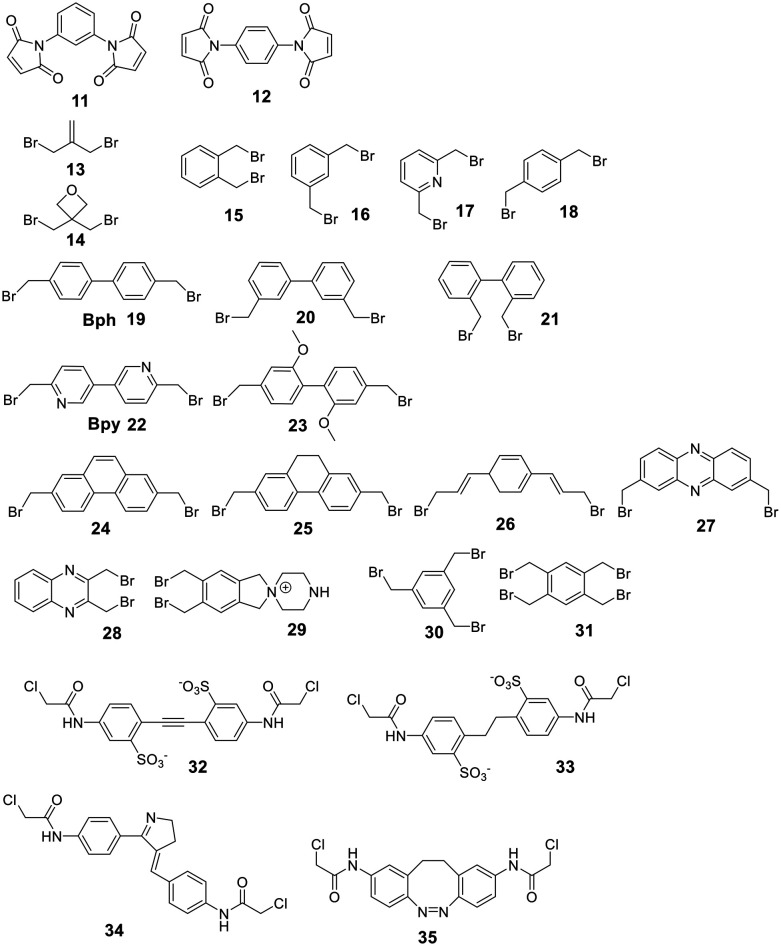
Examples of scaffolds used for thiol-to-thiol sidechain stapling.

Nucleophilic aromatic substitution and palladium-catalyzed arylation chemistry for peptide stapling was first introduced to react with the highly nucleophilic thiolate of cysteines (see below and Fig. 2) and was subsequently adapted to react with amine sidechains. As electrophiles for the nucleophilic aromatic substitution, perfluoroaryl-, perfluorodiphenylsulfone-, and dichlorotriazine-derived scaffolds are used.^75^ Installing an electron-withdrawing group at the *para* position of the electrophilic arene increases the S_N_Ar efficiency. All reactions proceeded in DMF and tris-basic or DIPEA-basic conditions on unprotected peptides (except for Cys, which would react faster).^75^ The lysine-aryl stapled peptides are stable under basic and oxidative conditions, in contrast to the Cys-aryl ones. On unprotected peptides, the palladium-catalyzed arylation of lysine is achieved in weak basic conditions and a preformed biarylphosphine-supported palladium(ii)-aryl complex (*t*-BuBrettPhos).^76^ Arg, Gln, Asn, the C-terminal amide, and the N-terminal primary amine are not compatible and lead to diarylation. The side reactions, however, can be suppressed when the Pd complex is the limiting reagent. Using 1,2-bis(4-bromophenoxy)ethane (**10**) as a scaffold, the p53 peptide was successfully stapled at *i*, *i* + 4 and *i*, *i* + 7.^76^

## 3. Disulfide cyclization

Cysteine is the preferred amino acid for chemical transformations of linear peptides due to the high nucleophilicity of the thiolate.^77^ Disulfides are a common structural motif found in proteins and other natural compounds to stabilize tertiary structures and conformations. Therefore, the specific distances of disulfide bonds connected cysteines best suited for stabilizing α-helical (*i*, *i* + 7)^78^ and β-sheet peptide conformations were identified early on.^79^

Disulfide formation proceeds readily between two proximal thiolates in an oxidative environment, such as air, I_2_, DMSO, or H_2_O_2_. However, disulfides are inherently unstable in a reducing environment and towards nucleophiles, particularly other thiols (thiol exchange). To improve their stability, disulfide groups have been replaced with lactam, thioether, selenium, triazole or dicarba analogues, with most of these methods requiring significant modifications of the synthetic building blocks.

## 4. Thioacetal formation

**Scheme 14:**
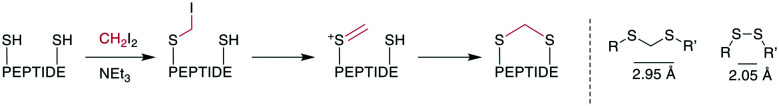
Left: reaction scheme with intermediates for the formation of methylene thioacetals from disulfides. Right: structural similarities between disulfides and methylene thioacetals.

The thioacetal as bridging motif has mostly attracted interest as a flexible, reduction-stable analogue for native disulfide bridges.^80–82^ The *S*–*S* distance in a methylene thioacetal is approximately 2.95 Å compared to 2.05 Å in a disulfide, and it maintains a similar flexibility and positions for the attachment points (Scheme 14, right).^80^ An early example of the formation of a methylene thioacetal was reported in 1999 by Ueki *et al.* when an enkephalin analogue with dimethylphosphinothioyl-protected cysteines was reacted with TBAF.^82^ This strategy was also employed for the synthesis of other pharmacologically relevant peptides, such as vasopressin^83^ and angiotensin II.^81^ Although the affinity for the angiotensin II (AT_2_) receptor decreased slightly, this nevertheless provided a 10-fold selectivity over the angiotensin I (AT_1_) receptor (ESI† Table S2).^81^

The convenient formation of thioacetals in an aqueous environment and under mild conditions without the need for protecting groups was made possible by a procedure described by Kourra and Cramer,^80^ which resembled harsher, previously reported reaction conditions for the formation of methylene thioacetals and, more generally, dithioethers.^84^ When thiols, or *in situ*-reduced disulfides, were reacted with diiodomethane and a base, they formed a methylene thioacetal with good yield. Mechanistically, one thiol replaces an iodide on CH_2_I_2_. Subsequently, the second iodide is eliminated, and the second thiol adds to the sulfonium ion (Scheme 14, left). The method proceeded with good yields for several peptide hormones and increased the reductive, serum, pH, and temperature stability while maintaining the affinity of oxytocin. Typically, the disulfide peptide is first reduced with TCEP, and the thioacetal is subsequently formed with 2.5–10 eq. of CH_2_I_2_ and 5–15 eq. of NEt_3_ in H_2_O/THF at room temperature over several hours. Encouraging results were obtained when this methodology was applied to insulin,^85^ the 58-residue protein bovine pancreatic trypsin inhibitor,^86^ adrenomedullin analogues,^87^ peptide mimetics binding to the HIV *trans*-activation response RNA,^88^ and in the chemical synthesis of the protein interleukin-2.^89^

## 5. Thioether formation

**Scheme 15:**
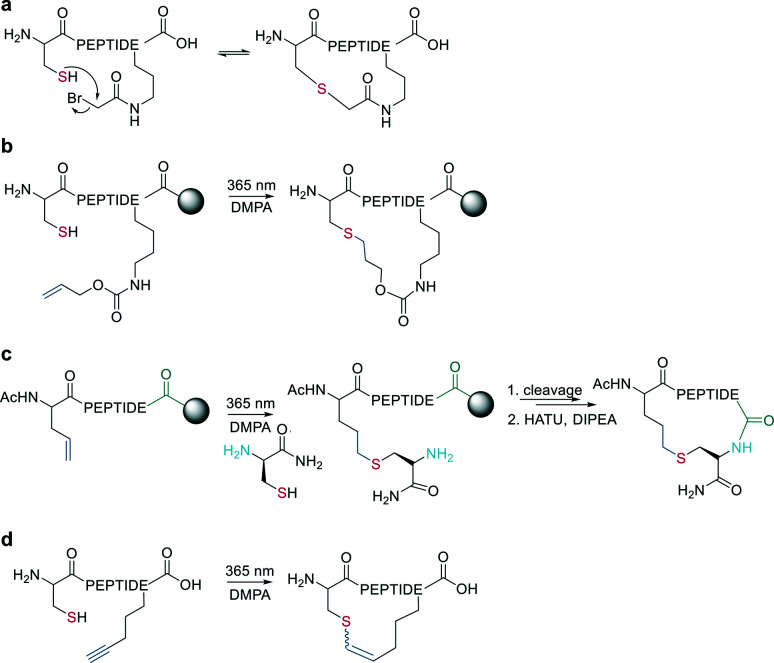
Macrocyclization *via* thioether formation; DMPA = 2,2-dimethoxy-2-phenylaceto-phenone. a: Reaction of thiol with bromoacetate-moiety. b: Thiol–ene reaction in solution. c: Thiol–ene reaction on-resin. d: Thiol–yne reaction.

The nucleophilicity of a cysteine thiol can be further exploited in sidechain-to-sidechain stapling through thioether formation. This has been applied to induce α-helicity in peptides by employing bromo- or chloroacetate as a reactive moiety, which can be coupled to a sidechain (*e.g.*, ornithine,^90^*O*-[2-bromoethyl]-tyrosine^91^) or the N-terminus (applied using mRNA display).^92^ Thioether formation occurs in aqueous buffers at pH 8 (Scheme 15, a).

Wang *et al.* applied the thiol–ene coupling to phage display screening by incorporating an electrophilic noncanonical amino acid (*N*ε-acryloyl-lysine) using amber suppression.^93^ A thioether bond for cyclization can also be generated by a radical addition of the thiol group to an alkene (*e.g.*, allyloxycarbonyl protecting group^94^) in a thiol–ene reaction on solid support. The radical reaction can be initiated in the presence of ultra-violet light irradiation and a radical initiator (Scheme 15, b). Similarly, Tian *et al.* used a radical thiol–ene to react a cysteine thiolate with an alkene-containing noncanonical amino acid. The final cyclization of the peptide was achieved by conventional amide formation between the amine of the N-terminal Cys and the C-terminus (Scheme 15, c).^95^

Similarly, vinyl sulfonamides can react with cysteines as Michael acceptors for on-resin cyclization. The vinyl sulfonamide is introduced by an N-terminal coupling of the commercially available reagent 2-chloroethane sulfonyl chloride.^96^ Thiol–yne coupling can also be employed for thioether-cyclization by reacting an alkyne-containing amino acid with cysteine under photo-induction, yielding mixtures of *E*- and *Z*-isomers (Scheme 15, d).^97^ Thioethers are also formed by generating a dehydroalanine and subsequent Michael-addition with a thiolate.^98,99^

### 5.1 Scaffold thioether formation

Secondary structures can be stabilized by reacting two cysteines, which are usually first reduced with mild reagents such as TCEP, with organic scaffolds. In some examples, this stabilization has resulted in an increase in cell permeability.^100^ Several electrophiles have been used for the cysteine scaffold cyclization reactions (Fig. 2). The first was reported by Kemp *et al.*, who showed that a β-sheet in a cyclic nonapeptide containing three cysteines could be stabilized using tribromomethylenebenzene (**30**).^101^ The nucleophilic substitution of bromomethylenearyl compounds is fast and chemoselective for cysteine in aqueous, mildly basic solutions (*e.g.* MeCN/NH_4_HCO_3_, DMF/DIPEA at pH 7.8–8.5), with a high conversion at room temperature,^102^ enabling the stapling of unprotected peptides.^16,17,101,102^

To achieve maximal stabilization of the secondary structure, Woolley *et al.* underlined the importance of a matching scaffold length and the distance distribution of attachment points, suggesting that enhanced rigidity improves the helicity.^103^ The bromomethylenebenzene scaffolds (called CLIPS, **15–18**) have been used extensively to stabilize helicity by stapling cysteines at positions *i*, *i* + 4.^102^ Additionally, bisarylmethylenebromide scaffolds (Bph **19**, Bpy **22**)^100^ are suitable for a rigid *i*, *i* + 7 (9–13 Å) configuration, and water-soluble schaffold **29** (ref. 103) is used for *i*, *i* + 11 (14–20 Å) stapling. Aliphatic scaffolds, such as 3-bromo-2-(bromomethyl)prop-1-ene (**13**), have also been reacted with cysteine thiolate to fix secondary structures (*e.g. i*, *i* + 7 for a rigid, folded backbone). This introduction of the isobutylene scaffold also improved passive membrane permeability and plasma stability.^104^ Similarly, the bisbromo-oxetane (**14**) was used to staple a secondary structure and improved important drug design parameters, such as solubility, basicity, lipophilicity, and metabolic stability.^105^

Some scaffolds can also be used to add functionality to the peptide during the cyclization step. For example, dichloroacetone adds a ketone moiety to the macrocycle, which could serve as a handle for other modifications.^106^ Another approach reacted tribromomethylenebenzene (**30**) monovalently with a wide variety of groups, including biotin, cholesterol, arachidonic acid and carboxyfluorescein, before cyclizing the unprotected peptide with the functionalized scaffold.^107^ The same approach has been used for the bioconjugation of proteins and antibodies,^108^ though a major drawback of bromomethylenearyl scaffolds is their limited solubility in aqueous solutions. Therefore, Smeenk *et al.* designed a bromomethylene scaffold (**29**) that combines improved solubility with the option for functionalization. Starting from a 1,2,4,5-tetrabromomethylenebenzene, derivatization with a 1,4-piperazine increases water solubility and offers a reactive amine to functionalize the scaffold.^109^

**Scheme 16:**
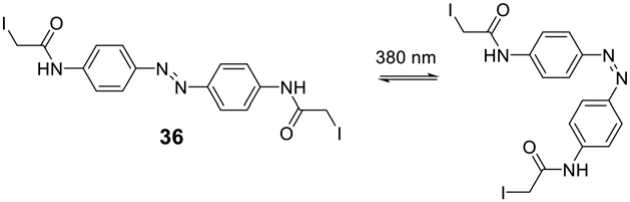
Thiol-to-thiol scaffold to generate photoswitchable peptides.

Cysteine-reactive scaffolds have also been used to introduce a photoswitch to cyclic peptides. Using an iodoacetamide-modified azobenzene scaffold (**36**), Woolley *et al.* reported a stapling method able to include α-helicity under photocontrol (Scheme 16).^110^ Another chromophore for photoswitching, benzylidene-pyrroline (**34**), confers a 10 Å change in end-to-end distance upon isomerization. The conformation of the target peptide can be switched from the *Z*-isomer (400 nm) to *E*-isomer (446 nm) in aqueous, neutral solutions. The *Z*-isomer has a slow thermal relaxation, permitting separation of the isomers by HPLC. By crosslinking cysteine sidechains in an *i*, *i* + 11 spacing, the *E*-isomer can stabilize an α-helix, while the *Z*-isomer is too short.^111^ Another example is the thiol-reactive chloroacetamido-substituted C2-bridged azobenzene (**35**) (407 nm, 518 nm), which caused model peptide FK-11 to undergo a helix-coil transition when cysteines at *i*, *i* + 11 were bridged.^112^

**Fig. 3:**
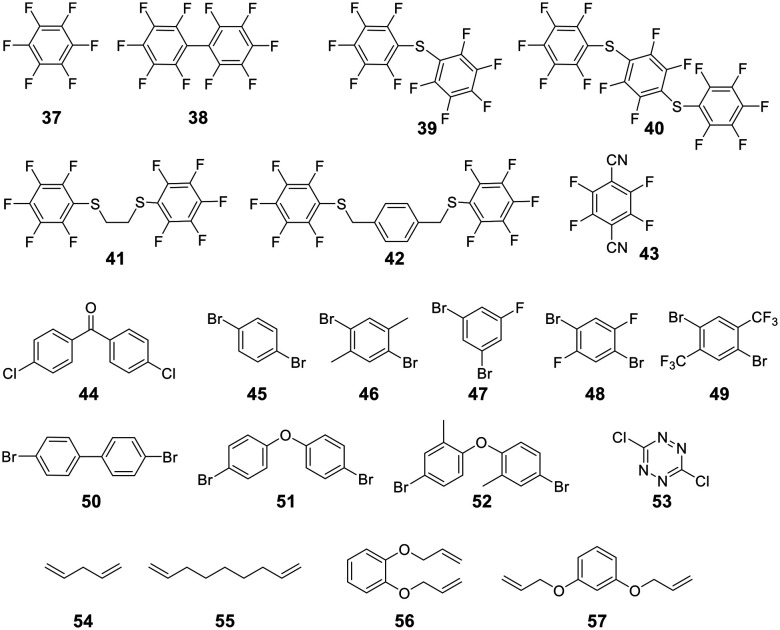
Frequently used scaffold for thioether-based stapling by 1,4-disubstition with perfluoro-scaffolds (**37–42**), Pd-mediated coupling with aryl dihalids (**44–53**) and thiol–ene reaction (**54–57**).

For linear peptides with more than two cysteines several scaffolds have been reported able to bridge multiple cysteines. For example, three cysteines can be bridged using 2,4,6-tris(bromomethylene)benzene (**30**), and four cysteines can be bridged using 1,2,4,5-tetrabromodurene (**31**).^102^ However, organic scaffolds with three or four spatially isometric thiol-reactive groups yield a mixture of regioisomers. 2,3,5,6-tetrafluoro-1,4-dicyanobenzene (**43**, Fig. 3) was proposed to reduce regioisomers due to a drastically changed reactivity of the remaining C–F sites after the first substitution, resulting in a stepwise crosslinking process.^113^ However, two regioisomers are still generated, except when bridging two cysteines and one penicillamine, where one specific bicyclic structure is yielded.^114^

Dichlorotetrazine was suggested as a reversible cyclization scaffold, which can be released by a photochemical trigger to generate thiocyanates and molecular nitrogen. The thiocyanates can be converted back to sulfhydryl groups (Cys) by reaction with cysteine. To tackle the low solubility of dichlorotetrazine in water, it was dissolved in chloroform and mixed vigorously with the peptide in phosphate buffer (pH 5).^115^

An emerging technique to promote cysteine stapling is the use of reactive aromatic linkers containing electron-withdrawing and activating moieties, such as perfluoroarenes (**39–44**,) that result exclusively in a 1,4-disubstitution.^116^ Increased helicity, stability, and cellular permeability can be obtained by stapling with perfluoroaryl scaffolds, and multiple scaffolds for different cysteine distances have been reported.^117^

Furthermore, aryl dihalides (**45–53**) have been shown to react with cysteines of an unprotected peptide in the presence of palladium under mild aqueous conditions (pH 5.5–8.5, small amounts DMF, DMSO, or MeCN).^118^ The careful choice of palladium ligand (RuPhos) led to a selective and fast C–S bond formation, though previous reports had shown that free thiols could inhibit palladium-catalyzed cross-coupling reactions^119^ and that Pd(ii) complexes could exhibit protease-like activity.^120^ This approach is chemoselective over serine, in contrast to palladium-mediated allylation,^121^ and the required bis-palladium crosslinking reagents can be generated in one-step from commercially available aryl dihalides.^122^ The *S*-arylated peptide was shown to be stable towards acids, bases, and thiol nucleophiles.^118^ However, cysteine-aryl homologues can be eliminated under basic conditions to form dehydroalanine or can be subject to oxidation.^75^

The thiol–ene reaction has also been applied for stapling and is especially suitable for certain bis-electrophilic linkers that are not sufficiently activated for *S*-alkylation, *e.g.* alkyl halides (**54–57**).^77,123^

Allyl sulfones enable site-selective cysteine coupling by reacting as a Michael-acceptor. Interestingly, the allyl sulfones can be used as a handle to introduce up to 3 different functionalities simultaneously.^124^ Another linker employing a Michael-addition reaction mechanism is 2,2-disubstituted cyclopentenedione, which also offers simultaneous derivatization and cyclization. The addition of a chaotropic agent increases the cyclization rate, though side reactions do occur, such as cysteine oxidation (SO_3_H), disulfide formation, and epimerization.^125^

**Scheme 17:**
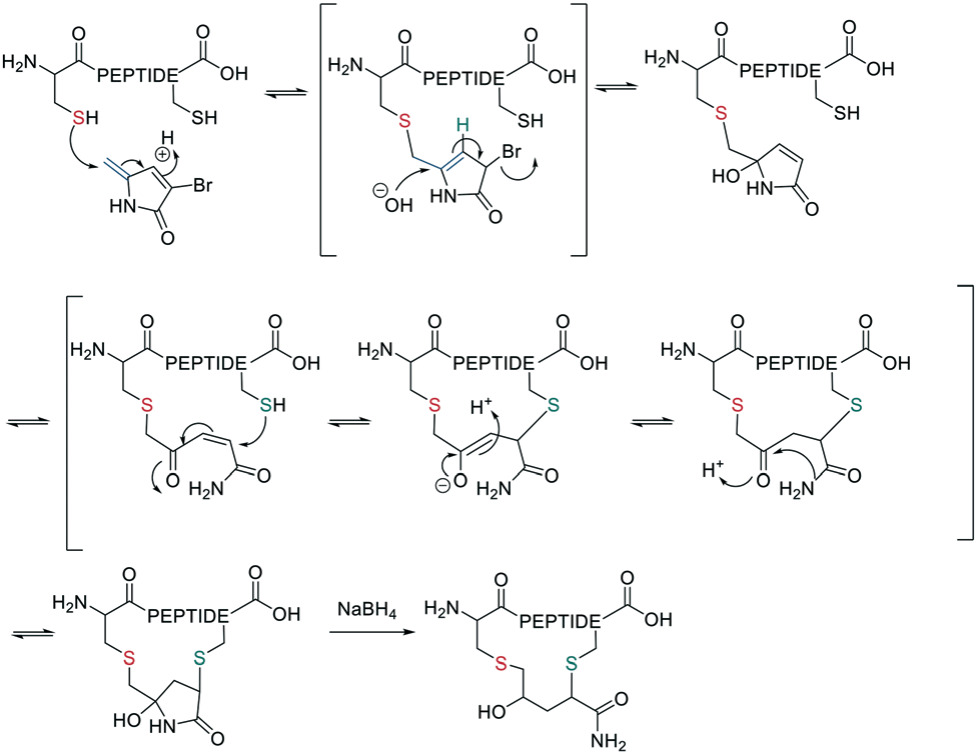
Thiol-to-thiol scaffold using 3-bromo-methylene pyrrolone.

**Scheme 18:**
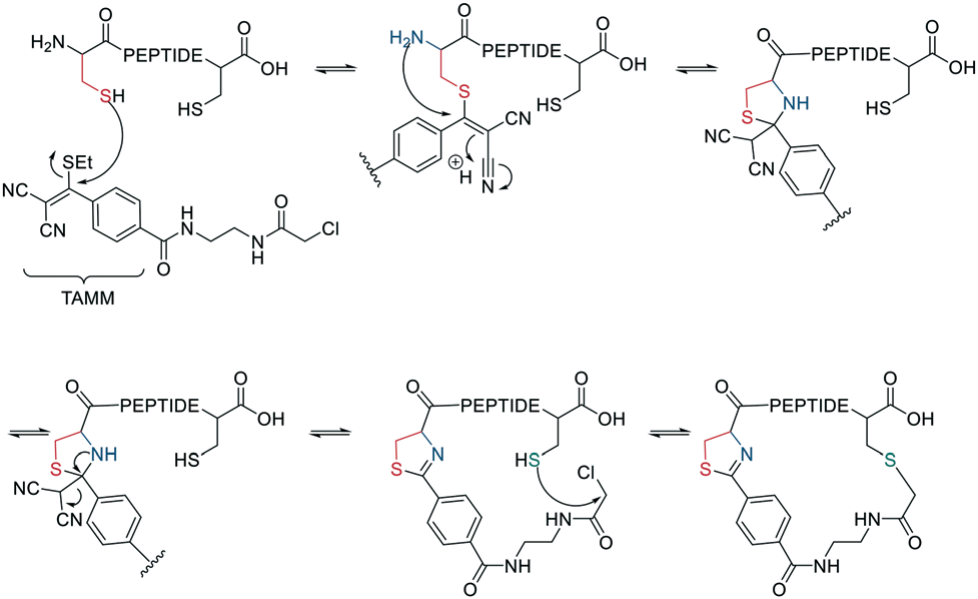
Reaction mechanism of cyclization generating a dihydrothiazole by reaction with TAMM (2-((alkylthio)(aryl)methylene)malononitrile).

Thiol–maleimide adducts are widely used for bioconjugation and peptide stapling,^126,127^ though the adducts decompose rapidly *via* hydrolysis and/or retro-Michael reactions. The addition of glutathione also reverses the stapling, which might find an application in targeted delivery.^127^ In a Mitsunobu-alkylation, the dibromo-maleimide can be further modified to introduce an alkyne as click handle.^127^ Zhang *et al.* have developed a maleimide derivative suitable as a scaffold for peptide stapling that is stable in aqueous solutions at pH 6–8.5 for multiple days.^128^ Its reaction with thiols in neutral aqueous solution yields high conversion within minutes and is highly specific for cysteines. The bridged scaffold can further be reduced with NaBH_4_ (Scheme 17) and can be synthesized with various functional groups (fluorescein, alkyne, biotin, and other).^128^ The reaction of 1,2-aminothiol with the thio malononitrile TAMM (2-((alkylthio)(aryl)methylene)malononitrile), which can be introduced to a Cys sidechain as a chloroacetyl, forms a thiazolidine, inducing an elimination of dicyanomethanide to afford a 2-aryl-4,5-dihydrothiazole (ADT). This reaction proceeds under biocompatible conditions (NaHCO_3_ or phosphate buffer, pH 7.4) (Scheme 18).^129^

4,5-Dibromo-1,2-dihydro-pyridazine-3,6-dione has been derivatized with TCEP to work as a reversible thiol-reactive scaffold.^130^ The reduction of disulfide bridges by the TCEP part could provide a high local concentration of the tethering group *in situ*.^131^

#### 5.1.1. Scaffold thioether reactions on *in vitro* selection systems

To obtain bicyclic peptides on disulfide-free gIII phage, Heinis *et al.* applied the tribromomethylenebenzene (TBMB, **30**) scaffold to cyclize a peptide library containing three cysteines. The reaction proceeded with 10 μM TBMB in 20 mM NH_4_HCO_3_, 5 mM EDTA, pH 8 for 1 h.^132^ The phages were still sufficiently infective, which enabled the phage display screening of bicyclic peptides. Further scaffolds suitable for building bicyclic peptides on phage by thioether formation have been reported.^133^ To increase the diversity of phage displayed libraries, linear peptides containing four cysteines were cyclized on phage with a bi-reactive scaffold to generate three isomers with different conformations per linear peptide sequence. Twelve different scaffolds have been applied.^134^ Similarly, mRNA-displayed peptides containing multiple noncanonical amino acids and two cysteines were also cyclized with dibromoxylene.^135^ Further scaffolds applied on phages are decafluoro-diphenylsulfone (**41**, Fig. 3)^136^ and 2,4-difluoro-6-hydroxy-1,3,5-benzenetricarbonitrile.^137^ The latter scaffold is soluble in buffer (pH 7.4), in contrast to previously reported perfluoroaryl scaffolds, and is chemoselective for cysteine in neutral conditions. It reacts with primary amines under basic conditions (*e.g.*, pH 9.2).^137^

### 5.2 Scaffold-mediated cyclization of thiol and amine

Scaffolds with two electrophilic groups have been used to bridge a cysteine thiolate with the N-terminal amino group.^138,139^ Kubota *et al.* introduced a stapling scaffold that can connect Cys and Lys sidechains on an unprotected peptide *via* Pd-mediated *S*-arylation and subsequent reaction of a tethered electrophile to the Lys sidechain.^140^ Another chemoselective cyclization on unprotected peptides generates isoindole-bridged cyclic peptides *via* the reaction of a lysine or the N-terminus and cysteine thiolate with *ortho*-phthalaldehyde (OPA) in aqueous buffer (PBS pH 7.4). The reaction yields a rapid and clean transformation that tolerates diverse functionalities. The exact reaction mechanism could not be identified, since trapping the imine with NaBH_4_ was unsuccessful. The isoindole moiety provides an option for further post-cyclization modifications.^141^ This approach was reported simultaneously by Todorovic *et al.*, who called it fluorescent isoindole crosslinking (FlICK), highlighting the built-in fluorescence. To alter the spectral properties, five modified OPA have been used.^142^

Luo *et al.* developed dinitroimidazole as a bifunctional and highly soluble (10 mM) scaffold that can react selectively with Lys or Cys sidechains, depending on the reaction conditions.^143^ In a wide pH range (pH 3.0–8.0), 1,4-dinitroimidazoles were cysteine-specific in aqueous solutions, while they modified Lys residues efficiently in organic solvents, such as dimethyl sulfoxide (DMSO) with weak bases through a ring-opening and ring-closing mechanism.^143^

## 6. Ether formation

Ethers could be an interesting peptide bridging motif because they are flexible, have multiple conformations, and are more stable than disulfides or thioethers to reduction, oxidation, or nucleophiles,^144^ yet there are limited examples of their use in peptide macrocyclization. Notably, biaryl ethers have played a role in the synthesis of many natural product peptides, in particular for antibiotic glycopeptides, such as vancomycin.^145–147^

Several methods can be used to obtain macrocyclic biaryl ether peptides, such as S_N_Ar reactions either in solution^145,148–151^ or on-resin,^152,153^ Ru-catalyzed reactions,^154^ strained ring openings by phenols,^155^ Ullman-type couplings,^156^ and Evans–Chan–Lam reactions.^147,157^ Similar to the formation of aryl ethers, Ru-catalyzed S_N_Ar reactions have also been employed in the syntheses of amines and thioethers.^158^ Generally, all these methods require custom-made building blocks and protecting group strategies to obtain a selective reaction.

**Scheme 19:**
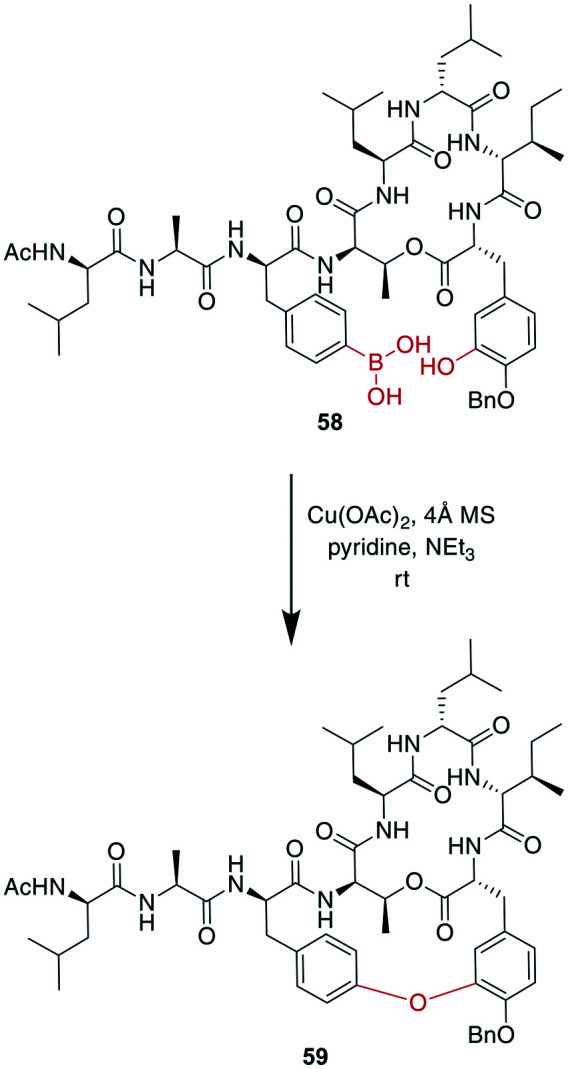
Reaction scheme for a macrocyclization step in the total synthesis of seongsanamide B using the Evans–Chan–Lam reaction.

**Scheme 20:**
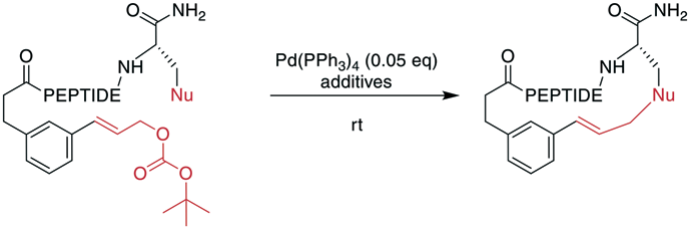
Application of the Tsuji–Trost reaction to the macrocyclization of peptides. If the nucleophile is a Tyr, a macrocyclic ether is obtained. Nu = nucleophile (phenols, amines, carboxylates, imidazoles), PEPTIDE = 2 or 3 amino acids.

A recent example is the total synthesis of the bicyclic depsipeptide seongsanamide B, for which Shabani and Hutton used a late-stage Evans–Chan–Lam reaction to form the second macrocycle. The required phenyl boronic acid was introduced as pinacol ester that was stable through amide coupling conditions and TFA-mediated cleavage from the solid phase, and it was liberated prior to the Evans–Chan–Lam reaction. This in-solution reaction gave the desired product **59** with a 26% yield (Scheme 19).^147^ Furthermore, the Tsuji–Trost reaction has been used on allylic esters and different native sidechains. In the absence of carboxylates, amines, histidines, or cysteines, the reaction is specific for tyrosine as a nucleophile. When amines or carboxylates are present, an excess of base is needed to form the more nucleophilic phenolate. Changing the catalyst from Pd(PPh_3_)_4_ to [PdCl(C_3_H_5_)]_2_ and using xantphos as a ligand yields a histidine-coupled product when no additional base is added, providing chemoselectivity when reaction conditions are tightly controlled (Scheme 20).^159,160^

**Scheme 21:**
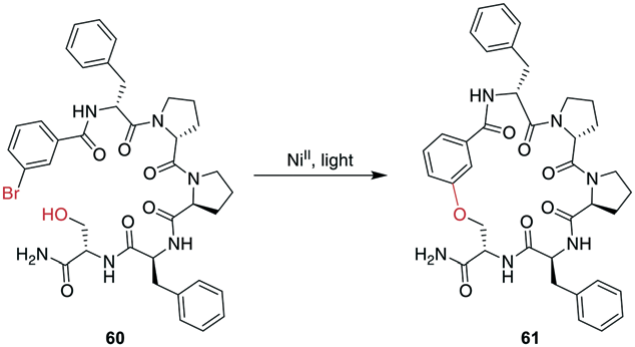
Ni^II^/photoredox-catalyzed ether formation between a bromo-benzoyl moiety and serine side chain.

In a remarkable example of how modern synthetic methods enable formerly hard-to-imagine bond formations, Lee *et al.* applied Ni/photoredox catalysis to the macrocyclization of peptides. To form an ether bond between a C-terminal serine and a 2-bromobenzoyl moiety at the N-terminus, they combined a Ni^II^-catalyst, 1,3-dicyano-2,4,5,6-tetrakis(diphenylamino)-benzene (4DAIPN), and irradiation with 450 nm light. However, if the C-terminus was an amide instead of an ester, it could react *via* the amide nitrogen instead of the serine sidechain (Scheme 21).^161^

To date, however, ether-containing cyclic peptides are predominantly accessed by other cyclization reactions, with the ether moiety introduced as part of a pre-formed building block.^144,162–165^

## 7. C–C single bond formation

### 7.1 Traditional cross couplings

Cross couplings, in which a new carbon–carbon bond is formed by transition metal catalysis, are one of the most important classes of reactions. In most of these reactions, a transition metal such as Pd inserts oxidatively into a carbon–(pseudo)halide bond, and subsequent transmetallation of an organometallic compound leads to two organic fragments bound to the catalyst. Formation of the new carbon–carbon bond occurs by reductive elimination. The various cross couplings differ mainly in substrates and catalysts, but all require both reaction partners to be pre-functionalized, excepting the Sonogashira coupling and Heck reaction.^166,167^

Most of the typical C–C cross couplings, namely the Suzuki, Stille, Negishi, Tsuji–Trost, Heck, and Sonogashira couplings, have been used to generate peptide macrocycles, although less often than other metal-catalyzed reactions, such as the copper-catalyzed azide–alkyne cycloaddition (CuAAC) or ring-closing metathesis (RCM).^160^ The use of the Suzuki coupling for amino acid modifications and peptides has been reviewed, including examples of macrocyclizations.^168,169^ Overall, it has been used in solution^170^ and on-resin to form five- and six-residue macrocycles.^171,172^

### 7.2 CH-Activation

In contrast to traditional cross couplings, couplings mediated by CH activation do not require the introduction of an organometal in the substrate, which is an important advantage as these usually strongly basic groups are problematic for peptides.^160^ In these reactions, one partner is a CH-group that typically reacts as a nucleophile with an organic halide, although more recent examples in which the CH group acts as an electrophile have also emerged. Typically, directing groups support the process by laying out the spatial arrangement and fine-tuning the electronic environment. The coupled CH-groups can be sp-, sp^2^- or sp^3^-hybridized centers, and for the latter group, β, γ, and δ modifications have been described.^173^ Despite the versatility of CH-activation, the reaction is usually performed on protected peptides, which contrasts with other metal-catalyzed macrocyclizations, such as RCM and CuAAC.^174–177^ CH activations on peptides, more generally as well as focused on macrocyclizations, have been reviewed recently.^160,173,178,179^

**Scheme 22:**
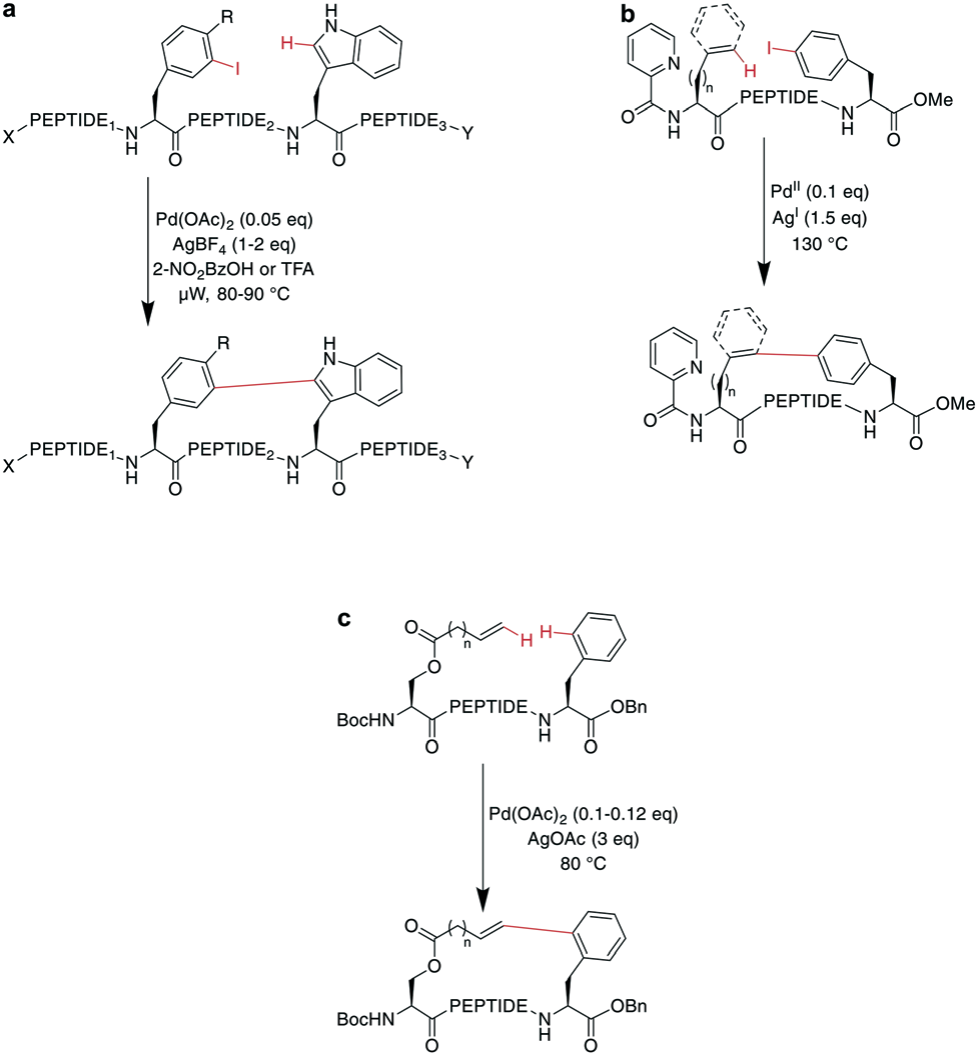
a: Peptides macrocyclized through their Trp-C2 and a iodophenyl residue from Phe or Tyr; X = H, Ac; Y = OH, NH_2_; R = H, OH; PEPTIDE_1_ = 0–1 amino acids: PEPTIDE_2_ = 0–3 amino acids; PEPTIDE_3_ = 0–1 amino acids; b: a typical substrate for a macrocyclization between a sp^2^-CH_2_ group and a phenyl iodide; *n* = 1, 2; PEPTIDE = 2, 3, 4 or 6 amino acids; c: oxidative cross-coupling between an alkenyl ester and a phenyl group to obtain macrocyclic peptides; *n* = 0, 2; PEPTIDE = 1–4 amino acids.

CH activation reactions can be used to selectively modify the C2 of the indole of Trp. These reactions have been used to successfully couple aryl halides to an assembled peptide using Pd-catalysis.^180^ In a series of intramolecular cross couplings between Trp and *m*-iodotyrosine and *m*-iodophenylalanine, Mendive-Tapia *et al.* achieved up to 100% conversions of linear precursors by linking positions from *i*, *i* + 1 to *i*, *i* + 5, though overall isolated yields were low (Scheme 22a). The cyclized peptides were stable against proteolytic degradation,^181^ and most amino acids, including His, Tyr, and Lys were tolerated, though Met was not compatible.^179,181^ Recently, Han *et al.* showed that this type of reaction can also be used to cross couple phenyl iodides with sp^2^-CH groups in the γ or δ position at the N-terminus when acylated with picolinic acid (Scheme 22b).^182^ Removing the necessity to introduce an aryl halide, the Wang group coupled phenyl residues, including on the phenylalanine sidechain, to terminal alkenes under Pd-catalysis with added AgOAc (Scheme 22c).^174,183,184^ Although most amino acids are tolerated in this reaction, sulfur-containing amino acids are not.^160^

**Scheme 23:**
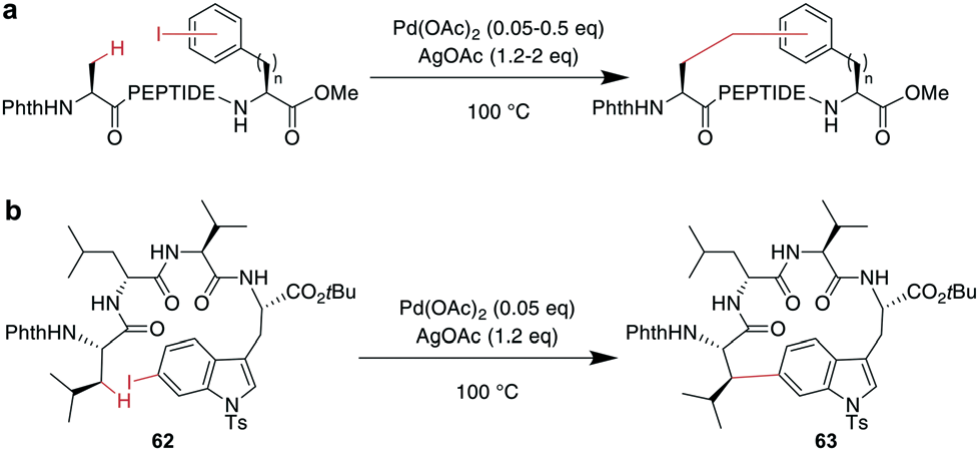
a: General reaction scheme for the oxidative cross coupling between β-Hs and *meta*- or *para* phenyl iodides; PEPTIDE = 1 or 2 amino acids; *n* = 1, 3. b: The application of the method to the A ring of celogentin C.

Phthaloyl-protection of the N-terminus leads to modified acidity which was employed to specifically activate the β-hydrogens on aliphatic sidechains to couple to aromatic halides under Pd-catalysis for macrocyclization (Scheme 23a).^175,176^ No epimerization occurs under the reaction conditions and cyclization between *i*, *i* + 4 and *i*, *i* + 3 residues was successful, but not for *i*, *i* + 2 residues. The reaction could be applied to the ring system A of the natural product celogentin C (Scheme 23b).^175,176^ Conveniently, the procedure could also be performed on-resin with continued C-to-N elongation after the cyclization and phthaloyl group removal. The products showed massively increased tryptic stability,^176^ and when applied to peptides with the integrin-binding motif RGD, binding to ανβ3 integrin-overexpressing cells was strongly increased (ESI† Table S2).^175^

**Scheme 24:**
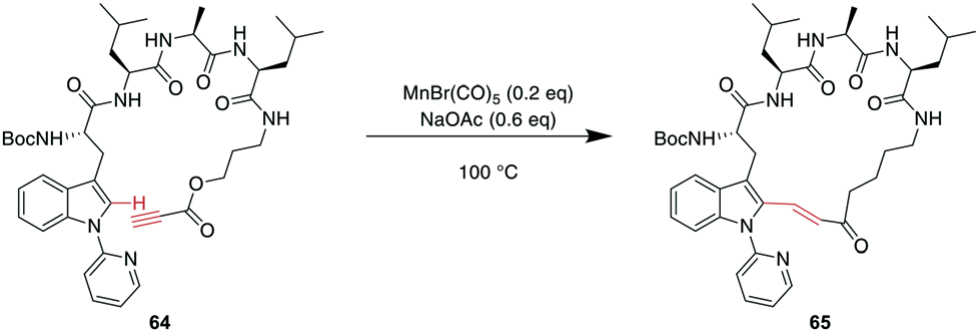
Propargylic esters can be hydroarylated with *N*-(2-pyridyl)-derivatised Trp under MnI catalysis to yield macrocyclic 3-indolyl acrylates.

More recently, transition metals other than Pd have been reported for CH activations. For example, Mn was shown to successfully alkylate the indole-C2 of *N*-pyridine Trp, where pyridine is essential as a directing group, providing an opportunity for selectivity when multiple Trp are present.^160^ Combining this CH-activation with the introduction of a propargylic ester in the peptide yielded α,β-unsaturated esters, which are versatile handles that can be further derivatized using, for example, cyclo- or conjugate additions (Scheme 24).^185^ Given the high temperatures generally employed for CH activations, this can also be achieved using Rh catalysis with acryl instead of propargylic esters with the addition of AgSbF_6_ and Cu(OAc)_2_ at 37 °C, an advantage for temperature-sensitive materials.^186^

### 7.3 Photocatalyzed reactions

**Scheme 25:**
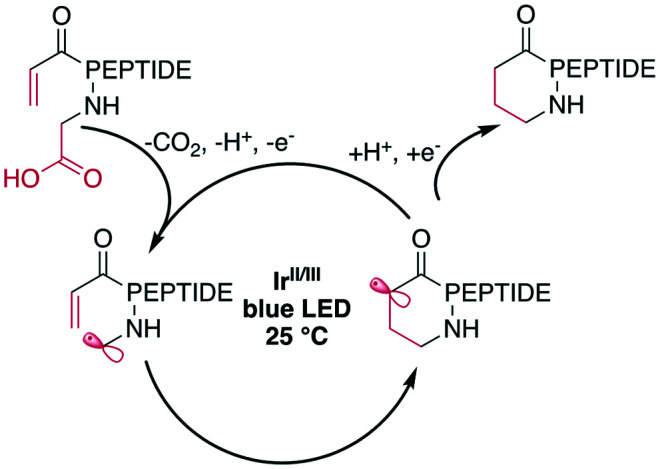
Simplified reaction mechanism for the Ir-photocatalyzed macrocyclization of *N*-acroyl peptides.

Peptide macrocyclization can also be performed using unfunctionalized iridium (Ir)- or other transition-metal-catalyzed photoredox reactions, which have been recently reviewed.^18^ Upon light irradiation, the Ir-catalyst promotes radical formation on the C-terminal carboxylate, leading to decarboxylation. The remaining carboradical undergoes a 1,4-addition with Michael acceptors, such as acrylates or malonates (Scheme 25). An impressive application of this methodology is the selective modification of the C-terminus of insulin, despite the presence of sidechain carboxylic acids and disulfides.^187^ The reaction was successfully applied to form rings ranging from 11 to 47 atoms in size from protected peptides and tolerated all tested residues, including His, Met, Arg, and Tyr.^177^ Similar efforts have been made using Ni-catalysts with phthalimide esters on the C-terminus.^160,188^

### 7.4 Glaser–Hay coupling

**Scheme 26:**
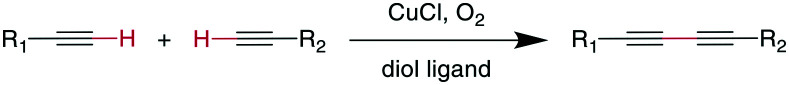
General reaction scheme for the Glaser–Hay reaction applied to peptides.

The Glaser reaction couples two alkynes to a dialkyne, creating a very rigid and extended bridging group (Scheme 26). This reaction occurs in the presence of O_2_ under Cu-catalysis, involving Cu^I^/Cu^II^ and possibly Cu^III^ oxidation states within the catalytic cycle.^189,190^ Modifications including Ni^2+^ salts have also been described, and the addition of diol ligands seems to be beneficial for oxidation-sensitive molecules, such as peptides, as the diols remove Cu^II^ species by gel formation.^190,191^ The reaction was employed successfully with *N*-, *O*-, and *C*-propargyl groups at *i*, *i* + 3 through *i*, *i* + 7 positions and could stabilize secondary structure motifs such as β-turns and α-helices.^192,193^ The resulting bis-alkyne can be reduced by catalytic hydrogenation.^191^

## 8. C–C double bond formation: alkene metathesis

Alkene metathesis, the reaction between two alkenes to form two new alkenes, was propelled into popularity in the 1990s by the development of chemoselective and stable molybdenum (Mo)- and ruthenium (Ru)-based catalysts.^194–198^ The reaction is most prominently applied to intramolecular reactions to generate cyclic systems, and labeled ring-closing metathesis (RCM) for this application. Its high tolerance for most functional groups as well as its usually high yields make it well suited for cyclizing relatively large, functionally diverse molecules. It has therefore been employed on a large number of complex structures, such as peptides and peptidomimetics. It can be performed in solution as well as on solid phase and has also been used in the synthesis of DNA-encoded libraries.^199,200^

**Scheme 27:**
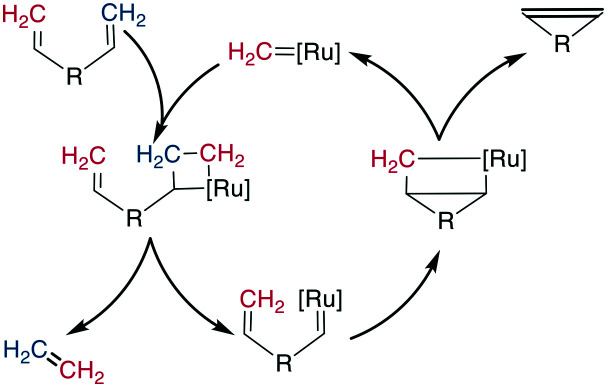
Reaction scheme for the Ru-catalyzed RCM.

Mechanistically, the catalyst-bound carbene or alkylidene first undergoes a [2 + 2]-cyclization with an alkene, leading to a metallacyclobutane intermediate. The subsequent ring opening can either revert unproductively to the starting materials or bind the catalyst to the substrate, which causes the release of another alkene (ethene for a terminal alkene substrate). This release is followed by a second [2 + 2] cycloaddition with the second alkene on the substrate, and finally, the formed metallacyclobutane opens to release the bridged product and the catalyst (Scheme 27). As all steps are in principle reversible, the reaction is under thermodynamic control such that the most stable product is the one predominantly formed, which is the *E*-alkene in an unstrained system. However, for macrocyclic peptides, mixtures of *E*/*Z*-alkenes are often obtained. By using two terminal alkenes the equilibrium is shifted towards the product side due to the released ethene gas, which is easily removed from the reaction mixture, leading to a high entropic contribution. However, in case of strained cyclic systems, RCM can still be challenging.^195^

**Fig. 4:**
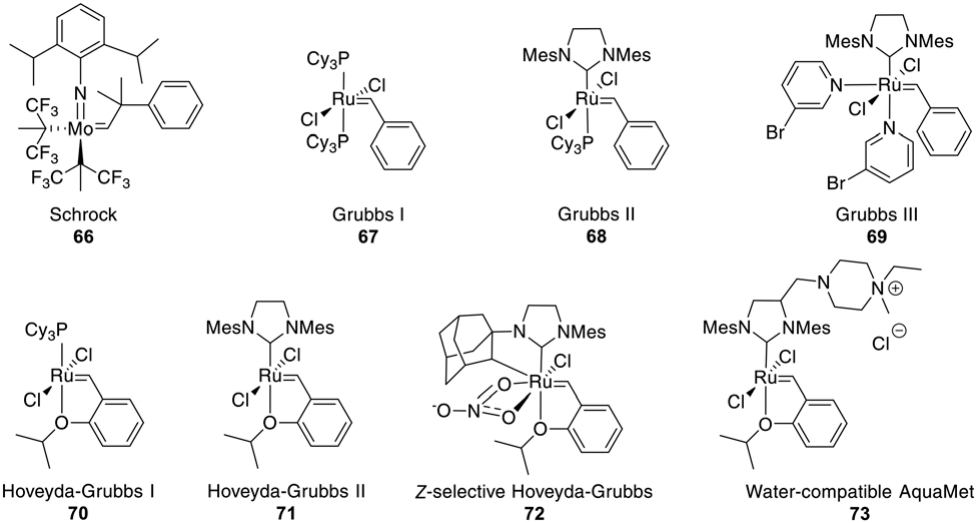
Frequently employed catalysts for metathesis reactions (Cy = cyclohexyl, Mes = mesityl = 2,4,6 trimethylphenyl).

The most common catalysts are Ru-based Grubbs **67–71** or, less frequently, Mo-based Schrock **66** catalysts. The Schrock catalysts are more reactive but less chemoselective and are water- and air-sensitive. Second (II) **68** and third (III) generation Grubbs **69** and Hoveyda–Grubbs **70–71** catalysts were developed to increase reactivity and thermostability (Fig. 4). For the Ru catalysts, unprotected amines are problematic though oxygen-bearing groups are tolerated, whereas the opposite is true for the Mo-catalysts.^195,197,201–203^

Typical rection conditions can vary substantially for different applications. Generally for peptide macrocyclizations, in-solution reactions tend to have lower concentrations (<10 mM), as high concentrations favor dimer over monomer formation, and catalyst loadings (<20 mol%). For on-resin reactions, MBHA or NovaPEG resins are considered superior to Wang and TentaGel, and the reaction is usually performed under microwave irradiation at elevated temperatures. A selection of reaction conditions can be found in the ESI† Tables S3 and S4, and publications containing comprehensive, general protocol descriptions are available.^204,205^

This reaction is among the most commonly applied to peptides for stabilizing secondary structures, in particular for α-helices.^206–209^ Furthermore, it has also been employed to stabilize or mimic other motifs, such as β-sheets,^210^ β-turns,^211,212^ polyproline II helices,^213^ 3_10_ helices,^214,215^ N-capping boxes,^216^ and disulfide bridges.^217,218^ The generated alkenes are generally more conformationally restricted than disulfides, but this is not always the case.^217^ Usually, the stapled peptides display improved proteolytic stability and cell permeability,^208,219,220^ with some exceptions.^221^ The effect on affinity, either through a *de novo* staple or when using it as surrogate for another functional group, is highly dependent on the specific peptide-target interaction as well as the alkene stereoisomer (*E*/*Z*) and stereochemistry, typically requiring an empirical approach.^13,217,218,222,223^

A successful example of the application of RCM to a peptide was provided by Song *et al.* Here, the affinity of a peptide sequence based on the natural binding partner of initiation factor eIF4E could be increased six-fold (ESI† Table S2).^223^ Furthermore, van Lierop *et al.* developed an insulin analogue in which the A6-A11 disulfide was replaced with an alkene. The *cis*-alkene analogue maintained affinity to the insulin receptors and showed improved efficacy in mice, whereas the *trans*-alkene had a 50-fold reduced binding affinity (ESI† Table S2).^224^ The application of RCM to peptides has been reviewed previously,^209,219,225^ including a perspective discussing all-hydrocarbon-stapled α-helical peptides in general.^208^

Alkenes for peptide stapling have been introduced as modified sidechains on carbon^13,204,226–230^ as well as on the α-*N*,^231–234^ side-chain aliphatic alcohols^206,229^ and phenols,^235,236^ C-terminal or side-chain acids,^46,237,238^ N-terminal carbamates,^237^ and cysteine thiol groups.^239^ Often, when the alkene is introduced as a modified C-bound chain, α-methyl-α-alkenyl sidechains are used for additional helix stabilisation.^13^

Depending on the strategy, it can be advantageous to have access to *Z*- and *E*-isomers in one step. These can mostly be separated by HPLC,^218,240,241^ though occasionally no separation is achieved.^242^ Ru-Catalysts selectively forming *Z*-alkenes **72** have been developed (Fig. 4), including some for more challenging substrates, such as those containing steric hindrance or polar groups near the reaction center.^242,243^ Available strategies for *Z*-selective RCM have been recently reviewed.^244^ Substrates undergoing selective *E*-alkene formation have been reported, such as those containing α,α-disubstituted amino acids between the bridging amino acids.^214^

**Scheme 28:**
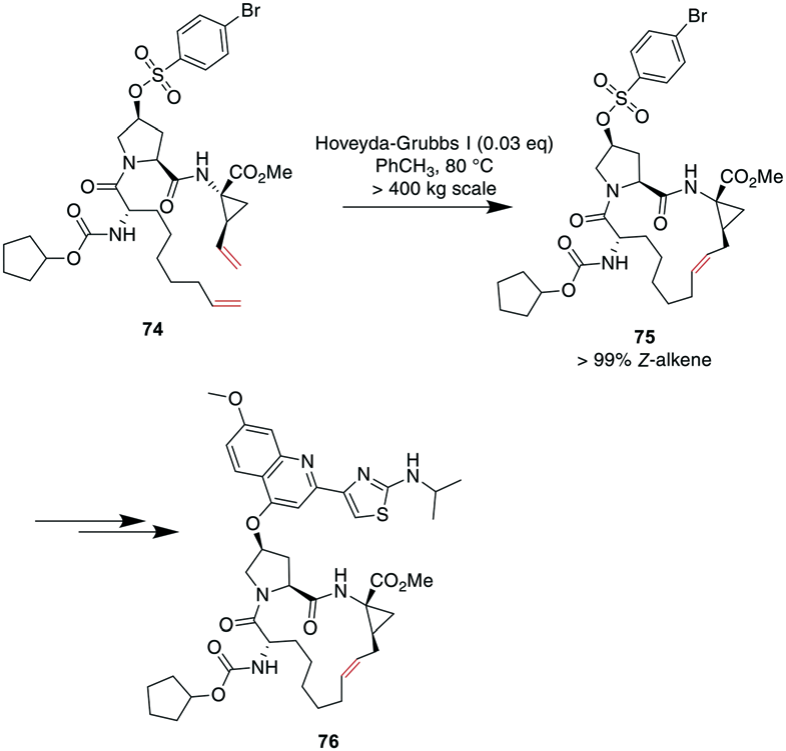
Synthesis of peptidomimetic **76** on an industrial scale with RCM as a key transformation for closing the macrocycle.

Demonstrating the impact of this reaction, chemists at Boehringer Ingelheim used RCM on a large industrial scale for the formation of a 15-membered macrocycle to produce an anti-hepatitis C peptidomimetic **76** (Scheme 28).^202,245,246^ Notably, when switching from a Grubbs I **67** to a Hoveyda–Grubbs I **70** catalyst, the reaction rate decreased but exclusively yielded the desired product **75** without concomitant isomeric or epimerized compounds.^245^ In contrast, switching to a Hoveyda-Grubbs II catalyst **71** drastically accelerated the reaction yet also increased the amount of dimers, emphasizing that the optimally balanced catalyst needs to be chosen carefully for specific reactivity requirements.^246^

**Scheme 29:**
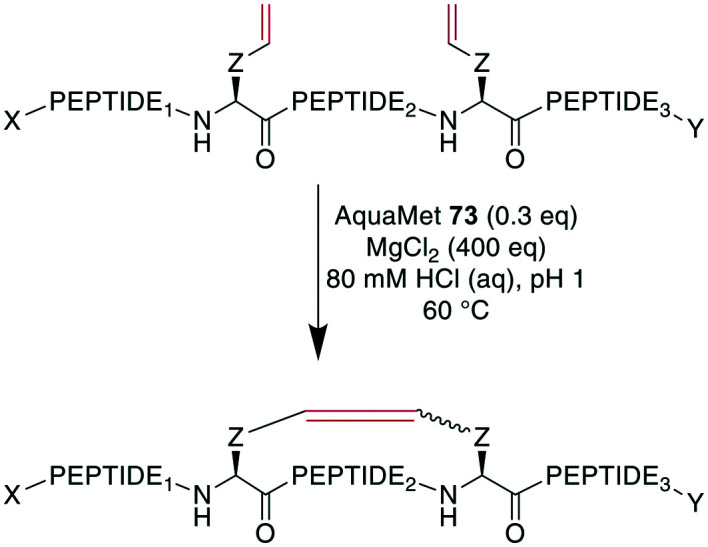
Synthesis of unprotected alkene-bridged cyclic peptides under aqueous conditions with AquaMet **73** as Ru-catalyst. AA = amino acid, X = H, Ac; Y = OH, NH_2_, Z = CH_2_, (CH_2_)_3_, CH_2_SCH_2_, PEPTIDE_1_ = 1 or 5 amino acids, PEPTIDE_2_ = 2 or 5 amino acids, PEPTIDE_3_ = 1 amino acid.

To demonstrate the flexibility of RCM, Gleeson *et al.* exploited the fact that Brønsted acids can mask free amines by protonation. They applied RCM to otherwise unprotected oxytocin, octreotate, two α-conotoxins, and an insulin fragment. The choice of solvent here was crucial, as the conversion of oxytocin proceeded quantitatively in DMF and with 84% and 66% conversion in MeOH and EtOH, respectively, while no product formation was observed in DMSO, MeCN, or solvent mixtures containing water.^247^ Cochrane *et al.* showed that the cyclization of unprotected peptides through allyl cysteinyl residues in solution with *t*BuOH/H_2_O as the solvent could be achieved by adding 5000 eq. of MgCl_2_,^239^ which was thought to act as a mild Lewis acid to effectively block potential peptide coordination sites to the Ru catalyst.^200^ Combining those previous findings, Masuda *et al.* were able to perform RCM on an unprotected model decapeptide in aqueous medium using the water-soluble Ru catalyst AquaMet **73**.^248,249^ The use of either acidic or neutral conditions in water or phosphate buffer containing MgCl_2_ allowed the synthesis of an octreotide analogue from different alkenes in yields of 53–64%. Amine-containing buffers were not tolerated, and the addition of a chaotropic agent such as guanidinium·HCl improved the yields substantially in neutral conditions (Scheme 29). Importantly, changing the peptide sequence decreased yields under neutral conditions. Conversely, the acidic conditions proceeded consistently satisfyingly with yields from 48–81%, suggesting the broader scope of these conditions.^249^

**Scheme 30:**
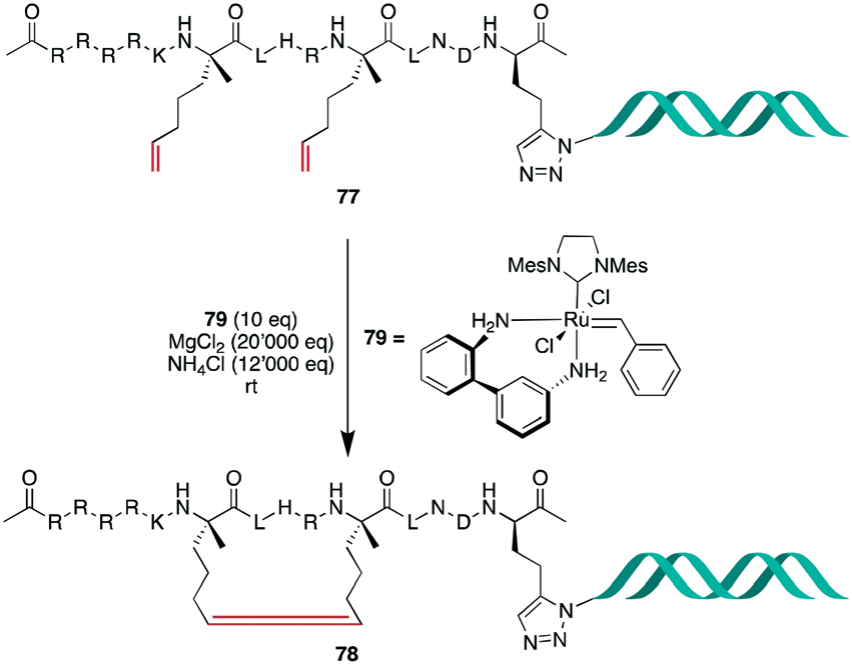
Synthetic scheme for the synthesis of an alkene-bridged α-helical cyclic peptide with a DNA tag.

Based on this work, Monty *et al.* embarked on the challenging task to optimize RCM for DNA-encoded libraries. Rationalizing that previously reported conditions^199^ could be further improved by maintaining acidic conditions to mask coordinating groups in the substrate and that improved solvent composition was needed to prevent phase separation between *t*BuOH and high ionic strength water, NH_4_Cl was added to the reaction conditions, and a mixture of H_2_O : EtOH : MeOAc (5 : 4 : 1) was used as the solvent. Diverse sets of simple substrates were tested, and generally robust conversion could be observed, although certain functional groups were not or poorly tolerated, such as 1,1-substituted alkenes, pyridines, and sulfonamides. Finally, they could obtain an α-helical stapled peptide **78** with 52–65% conversion, depending on the linker length between the peptide and DNA tag (Scheme 30).^200^

## 9. C–C triple bond formation: ring-closing alkyne metathesis

**Scheme 31:**
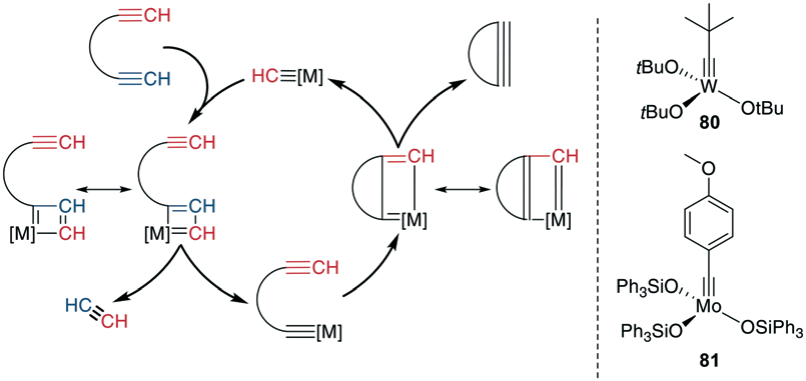
Reaction mechanism for the RCAM (left) and commonly used catalysts (right), [M] = metal catalyst.

The tungsten (W)- or Mo-catalyzed ring-closing alkyne metathesis (RCAM) produces a new alkyne in a similar fashion to alkene metathesis. An obvious difference to RCM is the resulting extended conformation and lack of isomers. The employed catalysts are high-valency W- and Mo-based complexes, particularly (*t*BuO)_3_W≡CtBu **80** and (Ph_3_SiO)Mo≡CPhOMe **81** (Scheme 31, right).

Mechanistically, an alkyne is first [2 + 2]-cycloadded to the catalyst-bound alkylidene to form a metallacyclobutadiene. The complex undergoes ring opening, binding the substrate to the catalyst. Upon cycloaddition of another alkyne and formation of a new metallacycle, the product is liberated after another ring opening (Scheme 31, left). Alkyne metathesis in general has already been comprehensively reviewed by Fürstner.^250^

Despite the similarities, the application of RCAM to peptides is less widespread than RCM.^251–255^ An early application of RCAM to a peptide cyclization of the A, B, C and E rings of nisin was published in 2005 by Ghalit *et al.* The N- and C-terminal-protected tetra- to heptapeptides were cyclized in solution using (*t*BuO)_3_W≡C*t*Bu **80** as a catalyst with 18–82% yield, with smaller cycles producing better results.^253^ In a further application, Cromm *et al.* synthesized bicyclic inhibitors against Rab8, a GTPase, using a combination of RCM and RCAM. The alkynes were successfully installed at position *i*, *i* + 3, *i*, *i* + 4, and *i*, *i* + 7 with (Ph_3_SiO)Mo≡CPhOMe **81** as the catalyst, yielding compounds with improved affinities (ESI† Table S2). Nicely, the work also demonstrated that RCM and RCAM could be performed selectively with both alkynes and alkenes present and in both possible orders.^254^

The advantages of introducing alkynes include the possibility to further modify those, such as to *Z*-alkenes using Lindlar's catalyst,^256^ to *E*-alkenes by hydrosliylation,^257^ or to dibromoalkenes with CuBr_2_.^254^

## 10. Triazole formation

### 10.1 1,4-Disubstituted triazole formation: the copper-catalyzed azide–alkyne cycloaddition

**Scheme 32:**
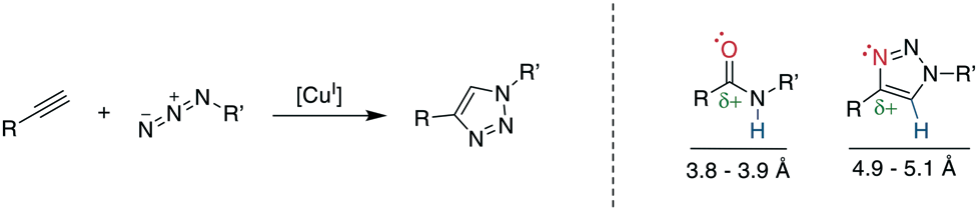
Left: reaction scheme of the CuI-catalyzed azide–alkyne cycloaddition. Right: comparison of electronic and steric properties of the *Z*-amide and the 1,4-substitued 1,2,3-triazole. Red: H-bond acceptor position, blue: H-bond donor position.

The copper (Cu)-catalyzed azide–alkyne cycloaddition (CuAAC) is an advancement of the Huisgen 1,3-dipolar cycloaddition and is the exemplary “click reaction” as established by Kolb, Finn, and Sharpless in 2001.^258^ Cu^I^-Mediated catalysis provides the robust, selective, and water-compatible formation of 1,4-substituted 1,2,3-triazoles from a terminal alkyne and an azide (Scheme 32, left).^259,260^ The 1,5-regioisomer is accessible through Ru-catalysis (*vide infra*), whereas traditional Huisgen cycloaddition conditions at high temperatures tend to give mixtures of isomers.^261^ The resulting 1,2,3-triazole is metabolically stable and mimics a *Z*-amide in terms of position of H-bond acceptors and donors, though there are somewhat different distances for the substituents. Furthermore, the dipolarity of the triazole compares well to the amide's (Scheme 32, right).^262–264^ The 1,2,3-triazole has been used to stabilize secondary structures or other motifs, including α-helices^265,266^ 3_10_-helices,^262,267^ β-hairpins,^268^ and disulfides.^21,269^ The reaction has been used extensively for, among others, bioconjugation and materials science, with comprehensive reviews available.^12,263,264,270^

**Scheme 33:**
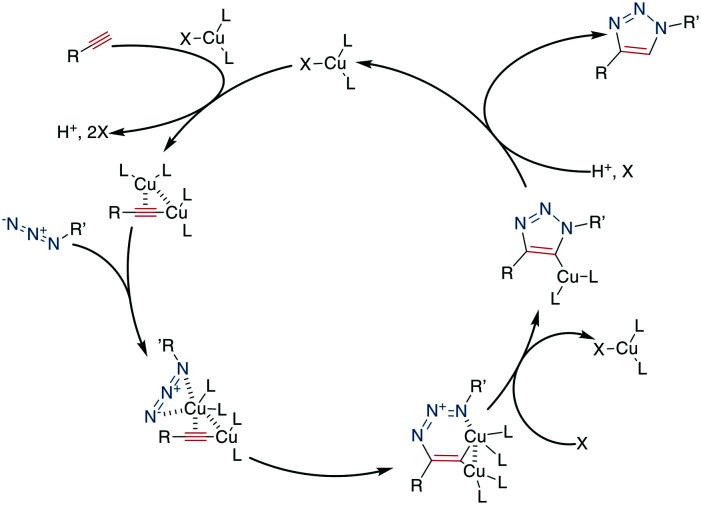
Reaction mechanism of the CuAAC, X: mostly SO_4_^2−^, I^−^, Br^−^, Cl^−^, PF_6_^−^, L = ligand such as THTPA, TBTA.

Despite its widespread usage, the detailed reaction mechanism has been challenging to establish. The current consensus^260^ is that the catalytic cycle is initiated by the coordination of the Cu^I^-species to the alkyne, followed by the tethering of the azide to the complex. The addition of the internal alkyne–carbon to the terminal azide–nitrogen forms a six-membered metallacycle. Reductive ring contraction and copper elimination releases the triazole (Scheme 33).

Many azides are commercially available, but can also be accessed by diazo transfer to primary amines, such as the ε-amino group of Lys.^271^ Likewise, alkynes are commercially available or can be synthetically accessed by, for instance, the Corey–Fuchs reaction or Seyferth–Gilbert homologation.^264^ When a short spacer on the C-terminus is desired for head-to-tail cyclization, it is possible to introduce a C-terminal propargylamine by using a silyl-based alkyne modifying (SAM)-resin.^272,273^

CuAAC does not require protecting groups and can be performed on-resin^267,271,274–278^ as well as in solution,^262,265–269,279–295^ which is more common and proceeds under mild conditions. Cu salts most frequently used are CuSO_4_ with sodium ascorbate, or Cu^I^ salts such as CuI and Cu(MeCN)_4_PF_6_. Where on-resin efforts fail, in solution approaches might still be successful, arguably due to the higher conformational flexibility of the starting material in solution.^282^

Cu species can oxidize His and Cys sidechains. In particular, Asp residues were shown to promote this by chelating Cu^II^ centers. The use of tris(triazolylmethyl)amine-based ligands, such as tris(3-hydroxypropyltriazolylmethyl)amine (THTPA) or tris[(1-benzyl-1*H*-1,2,3-triazol-4-yl)methyl]amine (TBTA), can substantially reduce this oxidative damage.^296^ Furthermore, by stabilizing the Cu^I^ species, the ligands accelerate the reaction.^297,298^ A selection of reported reaction conditions can be found in ESI† Tables S5 and S6.

Monitoring the reaction of an intramolecular CuAAC can be a challenge, as the starting material and product have the same molecular weight. To therefore indicate the reaction progress, one can use LCMS monitoring, the disappearance of the azide stretch at 2100 cm^−1^ in the IR,^287^ or a modified Kaiser reaction.^299^

As for other macrocyclizations, dimerization (*i.e.* an inter- instead of intramolecular reaction) is a major issue for the CuAAC,^300^ but can be reduced with higher Cu-concentrations^301^ and tends to be less problematic for on-resin-CuAAC of peptide macrocycles with less than six amino acids.^300^ Furthermore, for on-resin cyclization, this problem can frequently be addressed by changing the resin or solvent, particularly by using H-bond-disrupting solvents such as DMF or DMSO.^300,302,303^ Jagasia *et al.* extensively discussed the different parameters that affect the preference for mono- *versus* dimeric macrocyclization.^302^ Very recently, Kandler *et al.* performed an in-depth analysis of the parameters for on-resin CuAAC and found that when the macrocycle comprises six, seven, or eight amino acids, the monomeric form is predominantly obtained. DMF seemed to be the optimal solvent, and including 20% piperidine improved the monomer to dimer ratio.^303^

**Scheme 34:**
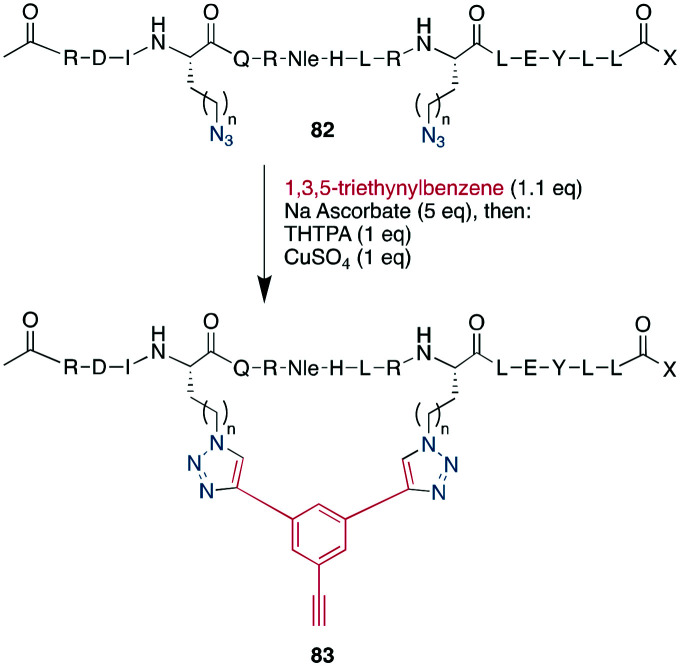
Performing CuAAC on helical peptides like **82** with triethynylbenzene allowed Tran *et al.* to additionally to stapling them to introduce further functionality on the cyclisation products such as **83**, *n* = 1–3.

As an example of CuAAC in peptide therapeutics, Gori used it to replace one of the two disulfides in the α-conotoxin MrIA, yielding compounds as efficacious as the native disulfides in a rat model for neuropathic pain while strongly increasing the plasma stability.^21^ By introducing two ω-azido amino acids within a peptide, the macrocyclization can also be done by two CuAACs employing a bis-alkynyl linker.^265,266,269,288–290,304^ This has been used as a stapling approach at the *i*, *i* +7 position for an α-helix^265,266^ and to successfully develop a peptidomimetic to allosterically target the kinase CK2, showing the potential of macrocyclic peptides even for a classical small-molecule target.^269^ This scaffold approach to bridge a peptide by a bis-alkynyl linker was further advanced by Tran *et al.* who used triethynylbenzene as linker. In their explorative study on the C-terminal α-helix from the Gs protein, this allowed them to introduce further functionality at the remaining free alkyne, such as a dye or biotin, after having stapled the peptides either at positions *i*, *i* + 7 or *i*, *i* + 9 (Scheme 34).^286^ Finally, the reaction was also used successfully in the synthesis of a DNA-encoded peptidomimetic library with 10^6^ members to identify ligands against several targets with *K*_D_s in the μM range.^291^ Showcasing the versatility of having a set of chemoselective reactions, several efforts have successfully obtained multicyclic peptides by combining different cyclisation strategies, such as CuAAC, enzymatically-mediated lactamization, oxime ligations, or thioether formation on scaffolds, yielding structurally unique moieties.^305–307^

### 10.2 1,5-Disubstituted 1,2,3-triazole formation: ruthenium-catalyzed azide–alkyne cycloaddition

**Scheme 35:**
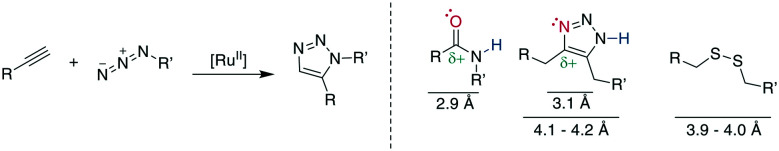
Left: reaction scheme for the Ru^II^-catalysed RuAAC, right: comparison of electronic and steric properties of the 1,4-substitued 1,2,3-triazole and the *E*-amide as well as a disulfide bond. Red: H-bond acceptor position, blue: H-bond donor position.

A 1,5-disubstiuted 1,2,3-triazole is similar to an *E*-amide bond in terms of H-bond acceptor and donor positions and can function as a bioisostere for it. Additionally, as the C_α_–C_α_ distance in a 1,5-substituted triazole formed between β-azidohomoalanine and propargyl glycine is similar to the C_α_–C_α_ distance in a cysteine disulfide (4.1–4.2 Å *versus* 3.9–4.0 Å), it has also attracted substantial interest as a disulfide mimetic (Scheme 35, right).^308–310^

**Scheme 36:**
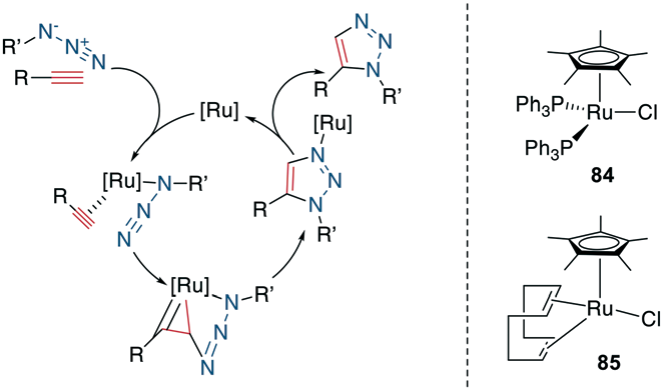
Left: mechanism of the RuAAC, right: structures of commonly used catalysts for RuAAC. [Ru] = ruthenium catalyst.

Similarly to the 1,4-disubstituted triazoles, their 1,5-disubstituted counterparts can be obtained regioselectively by a transition-metal catalyzed cycloaddition, although by Ru- instead of Cu-catalysis (RuAAC) (Scheme 35, left).^311^ In contrast to the CuAAC, internal alkynes can also undergo RuAAC, allowing the introduction of additional substituents.^311,312^ Here, the regioisomer obtained depends on the steric and electronic properties of the alkyne substituents.^312^ Common catalysts include [Cp*RuCl], Cp*RuCl(PPh_3_)_2_, **84** and Cp*RuCl(COD) **85** (Scheme 36, right), the latter reported to work particularly well for secondary azides^313^ and reacts at room temperature.^314^ With Ru-catalysis, carboxylic acids and sidechains need to be protected,^308,310,315^ though the reaction can still be used in solution^310,316–318^ as well as on solid support,^287,304,308,309,319^ usually with 15–50 mol% catalyst loading at elevated temperatures and in DMF. An overview of reaction conditions can be found in ESI† Tables S7 and S8.

Mechanistically, the catalytic cycle begins with the displacement of Ru-ligands and coordination of the azide and alkyne. Oxidative coupling forms a metallacycle *via* a new bond between the less sterically hindered, more electronegative carbon of the alkyne and the terminal nitrogen of the azide. After reductive elimination, this produces the 1,5-disubsituted 1,2,3-triazole for terminal alkynes (Scheme 36, left).^314^ Under high temperatures reminiscent of classical Huisgen conditions, the 1,4-substitution regioisomer can also be obtained.^316^ Shortly after its discovery, this reaction was already applied as a turn inducer in a peptoid^320^ and as a replacement of an *E*-amide in RNase A.^313^ It was later used as a disulfide substitute in the sunflower trypsin inhibitor 1 (SFTI-1).^321^

Multiple examples underline the higher bioisostery for disulfides of 1,5-sbstituted triazoles over 1,4-substituted triazoles. For example, the affinity was retained after the replacement of a disulfide in urotensin-II analogues by a 1,5-triazole, whereas the affinity was reduced or completely lost for 1,4-triazoles, which could be linked to structural reasons.^287^ Similar observations were made for SFTI-1 (ESI† Table S2).^308,310^ Further examples demonstrate the impressive potential of 1,5-disubstutited triazoles as disulfide isosteres by improving pharmacokinetic or creating changes in the pharmacodynamic profile.^304,309,319^

### 10.3 1,5-Disubstituted 1,2,3-triazole formation: alternative approaches

**Scheme 37:**
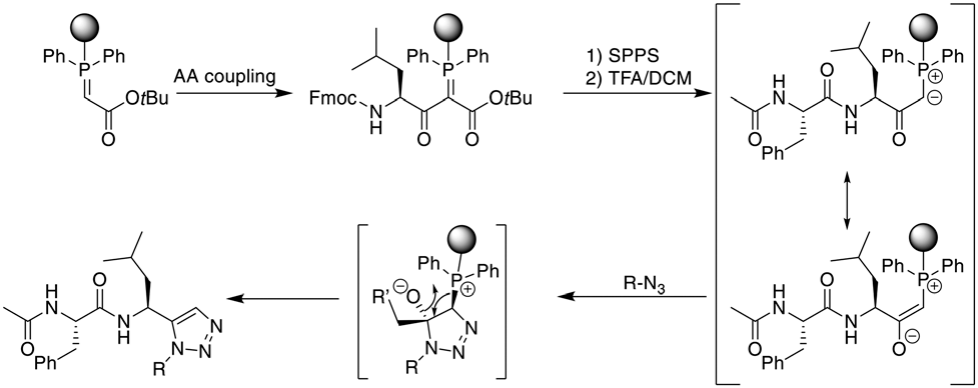
Synthetic scheme for the metal-free synthesis of 1,5-substituted triazole peptides. AA = amino acid.

As an alternative approach to the metal-catalyzed formation of 1,5-disubstituted triazoles, the Rademann group published an elegant method to introduce a 1,5-disubstituted 1,2,3-triazole on-resin. A resin-bound phophsphoranylidene acetate reacts with the carboxyl group of an Fmoc-amino acid, which can subsequently be elongated using standard SPPS. Treatment with acid liberates an α-carbonyl phosphorous ylide that, upon treatment with an azide, directly yields a 1,5-disubstituted triazole without metal-catalysis (Scheme 37).^322^ This was subsequently transferred to the on-resin cyclization of peptides. For dipeptide synthesis, only dimeric products were obtained, while tri- and tetrapeptides yielded mixtures of dimeric and monomeric products. A penta- and an octapeptide were obtained exclusively as monomeric products, making the method more attractive for larger macrocyles.^323^

## 11. Macrocyclization *via* multicomponent reactions

**Scheme 38:**
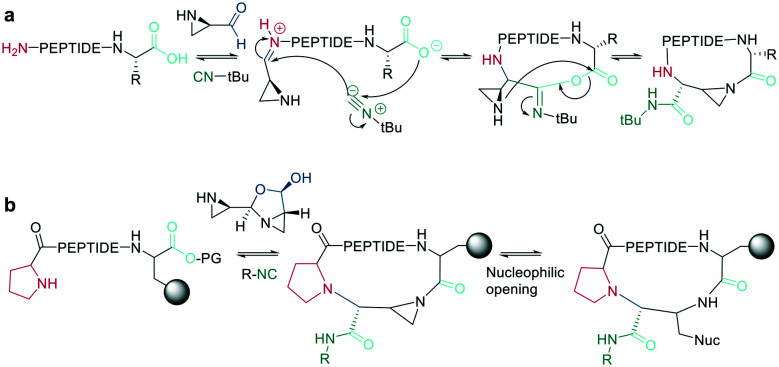
Mechanism of Ugi reaction using aziridine aldehyde a: in solution and b: on resin.

In recent years, the interest in multicomponent reactions for peptide macrocyclization is increasing and has been recently reviewed in detail.^324^ The Ugi reaction, which is a 4-component reaction combining isocyanide, amino, carboxylic acid, and aldehyde functionalities, yields *N*-methylated amides. The advantage of this reaction for peptide cyclization is the possibility of increasing molecular diversity during the ring closing step through the use of non-amino-acid building blocks. It was first applied in the context of peptide synthesis to obtain linear peptide esters^325^ and peptidomimetics,^326^ while cyclic peptides were generated though several subsequent Ugi reactions.^327^ The Ugi reaction has been used in the synthesis of head-to-tail cyclic peptides from linear peptides (amine and carboxylic acid) and conventional aldehydes. Due to the lack of diastereoselectivity^328^ and cyclodimerization, which can form the dominant product, low yields are achieved. The low selectivity can be explained by a kinetically competitive intermolecular process due to the slow transannular attack of the amine onto the mixed anhydride, which Hili *et al.* solved using *tert*-butyl-isocyanide and an aziridine aldehyde to obtain a cyclic piperazinone in high yields as a single diastereoisomer.^329^ With an amino aldehyde, the attack of the exocyclic nucleophilic aziridine is fast. A non-nucleophilic solvent, such as trifluoroethanol, prevents the premature solvolysis of the mixed anhydride (Scheme 38, a).^329^ For later bioconjugation, the activated aziridine ring within the cyclic peptide can be used *via* nucleophilic ring opening (Scheme 38, b).

Ugi-mediated head-to-tail cyclization was adapted to on-resin macrocyclization using side-chain attachment of the C-terminal amino acid and an orthogonal protecting group strategy (allyl ester) to selectively deprotect the C-terminal COOH on resin followed by Fmoc removal and reaction with an aziridine aldehyde dimer and *tert*-butyl isocyanide in equal parts DCM/TFE (Scheme 38, b).^330^

The Ugi reaction is also suitable for sidechain-to-sidechain and sidechain-to-tail/head cyclization, which was shown to be faster and more efficient than head-to-tail cyclization, probably due to the higher flexibility of the sidechains. By carefully choosing the isocyanide component, the diversity of the peptide scaffold can be increased *via N*-substitution of the newly formed amide.^331^ This approach was applied to stabilize secondary structures and simultaneously functionalize the sidechain-tethering lactam. To achieve this on-resin, a peptide was built using three dimensional orthogonal protecting groups for asparagine and lysine (alloc, allyl). Condensation of paraformaldehyde with pyrrolidine generated a pyrrolidinium ion, which is crucial for complete conversion to the imine by aminocatalysis mediated transamination, since on-resin imine formation is difficult to achieve with paraformaldehyde.^332^ Careful washing removes any remaining base before isonitrile is added to finally cyclize the peptide.^333^

**Scheme 39:**
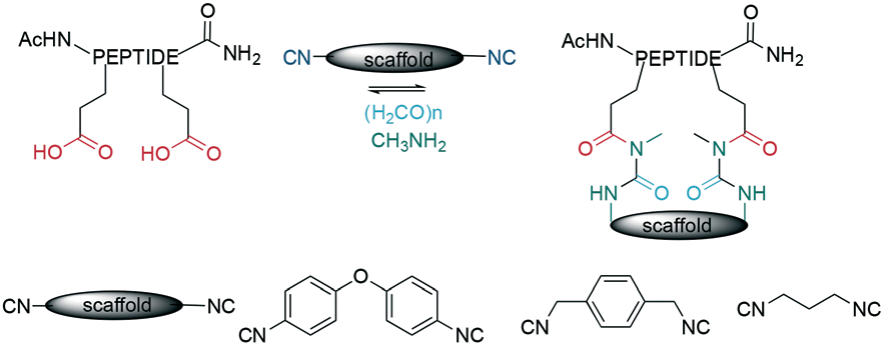
Synthetic scheme for the four component Ugi reaction.

As for many cyclization chemistries, the scaffold strategy has also been applied for Ugi multi component reactions (MCR), where linear peptides containing two acidic amino acids are coupled with diisocyanide scaffolds to generate sidechain-to-sidechain cyclized peptides (Scheme 39). Cyclization here is achieved in a pseudo-dilution protocol by slowly adding the peptide diacid and the diisocyanide to a mixture containing the preformed imine. However, this is slow, with reaction times of 96 h.^334^ A recent report highlighted the diversity achieved by distinct combinations of amino and isocyanide components, identifying stapled peptides that inhibited p53/MDM2/X.^335^

**Scheme 40:**
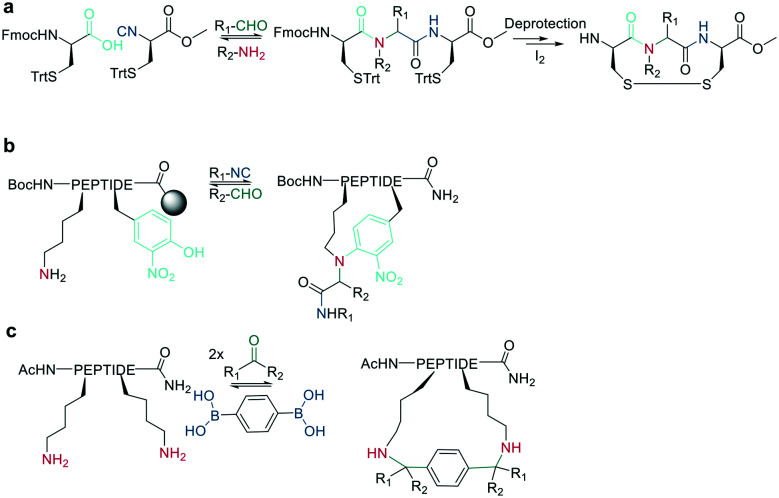
a: Sulfur-Ugi-switch reaction of an *in situ* assembled peptide with successive disulfide formation. b: Ugi-smiles reaction with nitro-tyrosine as acidic component. c: Petasis reaction for peptide scaffolding.

Macrocyclization *via* Ugi MCR can be combined with subsequent disulfide formation to generate bicyclic peptides.^336^ A similar approach called the sulfur-switch Ugi reaction was proposed to synthesize disulfide-linked cyclic peptides *de novo* from four components, followed by oxidative cyclization of the two cysteines with I_2_ to form disulfides (Scheme 40, a). The double mercapto input that is possible on each Ugi component yields six topologically possible combinations.^337^ To generate macrocyclic peptides in solution and on resin, the carboxylic acid in the classic four-component Ugi is replaced by an electron-poor phenol (such as 3-nitrotyrosine) in the Ugi-smiles reaction to yield tertiary nitroanilines (Scheme 40, b).^338^

The Petasis reaction, also known as the borono-Mannich reaction,^339^ was reported for late-stage diversification (on-resin) and stapling (in solution, Scheme 40, c) of peptides. This three-component condensation of an aldehyde/ketone, an amine (*e.g.*, Lys sidechain), and an aryl/vinyl boronic ester/boronic acid depends on imine formation *via* transamination, as described for the on-resin Ugi reaction above, and the addition of boronic acid. The outcome and conversion rate of the Petasis reaction depends on the reactivity of the boronic acid, leading to singly and doubly modified products. To achieve stapling in solution, pseudo-dilution conditions were used by the slow addition of boronic acid and peptide to a solution of oxo-component.^340^

**Scheme 41:**
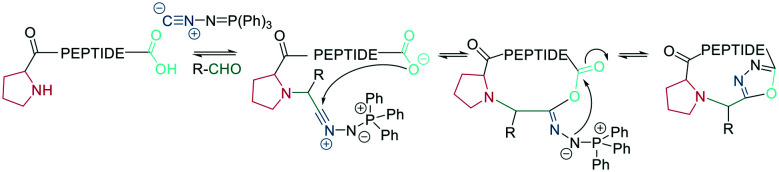
Multicomponent reaction for peptide macrocyclization employing an aldehyde and (*N*-isocyanimino)triphenylphosphorane to form a 1,3,4-oxadiazole.

A head-to-tail peptidomimetic can be generated in one step *via* the multicomponent reaction of an aldehyde, linear peptide, and (*N*-isocyanimino)triphenylphosphorane.^6^ The resulting backbone contains a 1,3,4-oxadiazole, which was shown to stabilize a unique intramolecular hydrogen-bond network and enable a high passive membrane permeability in contrast to the analogous homodetic macrocycles. Oxadiazoles have also been a focus of medicinal chemistry for being a proteolytically stable isostere of amides. The aldehyde component serves as a linker between the N-terminus, and oxadiazole and can mimic amino acid sidechains based on aldehyde substituents; for example, phenylacetaldehyde mimics phenylalanine and isovaleraldehyde mimics leucine. However, the reaction produces both diastereomers. For efficient cyclization, this macrocyclization approach uses a zwitterionic control element, which prevents oligomerization even for more constrained 4-*mer* sequences and at high concentrations (5–100 mM peptide). It was proposed that the positively charged triphenylphosphonium ion augments interaction between the chain termini, leading to a more efficient macrocyclization (Scheme 41).

## Conclusions

The favorable properties of peptidic macrocycles and peptidomimetics as potential drug leads have led to the rapid evolution of peptide chemistry beyond traditional amide formation. This is especially true for chemistries that expand the chemical space, such as derivatizing natural linear peptides with organic scaffolds, as these are expected to further improve the drug-like properties of peptides and peptidomimetics and extend the chemical space towards new therapeutic chimeric modalities. With more and more mild, specific, and mutually orthogonal cyclization methods at hand, the possibility for synthesizing highly conformationally constrained, more chemically diverse peptides and peptidomimetics in a controlled manner is continuously increasing. Given that modifying the peptide structure post-discovery can reduce the binding affinity compared to the initial hit, the increasing number of reactions compatible with *in vitro* selection systems and encoded combinatorial libraries will accelerate drug discovery efforts of those new macrocyclic peptidomimetic modalities, opening a great future for peptide-derived drug discovery.

## Author contributions

CB and CL have both conceptualized and written the manuscript.

## Conflicts of interest

There are no conflicts to declare.

## Supplementary Material

MD-012-D1MD00083G-s001
