# Supplementary material for: Macrocyclization strategies for cyclic peptides and peptidomimetics
Source: RSC Med Chem. 2021 Jun 29;12(8):1325–51. doi: 10.1039/d1md00083g (PMC8372203; doi:10.1039/d1md00083g)
Supplement: MD-012-D1MD00083G-s001 [file MD-012-D1MD00083G-s001.pdf]

Electronic Supplementary Material (ESI) for RSC Medicinal Chemistry.
This journal is © The Royal Society of Chemistry 2021

Macrocyclization strategies for cyclic peptides and peptidomimetics

Clément Bechtler[a] and Christina Lamers[a]*

Department Pharmaceutical Sciences, University of Basel, Klingelbergstr. 50, 4056 Basel, Switzerland.

Supplementary Table S1: Commonly used macrocyclization reactions for peptides. The required reaction partners are listed as well as if they have been reported to work succesfully on-resin, in solution, in a chemoselective way or on unprotected petides, a green tick signfiying it has been reported, a red cross that it has not been reported.

Bond or functional group formed	Required functional groups: Reaction partner I (N-terminus)	Reaction partner II (C-terminus)	Reaction	On-resin	In solution	Chemoselective	Unprotected	Comments
Amide	H_2N-	-COOH	Amide coupling	✓	✓	⍰	✓	
	HNCys-	-COSR	NCL	✓	✓	✓	✓	
	HNAla-	-COSR	NCL + desulfurisation	✓	✓	✓	✓	
	H_2N-	-COSR	aminolysis	⍰	✓	✓	Lys	Other lysine needs to be protected
	H_2N-	-COO-Sanger	aminolysis	⍰	✓	✓	Lys	Other lysine needs to be protected
	H_2N-	-COO-Dbz	aminolysis	✓	✓	✓	⍰	
	N_3CH_2NH-	$-COSCH_2PPh_2$	Staudinger	⍰	✓	✓	⍰	
	2-amino thioamide-	-COOH	Ag-promoted lactamisation	✓	✓	✓	✓	
	HNCys-	-glycolaldehyde	Thiazolidine formation	⍰	✓	✓	⍰	Or sidechain aldehyde
	RONH-	-Ketoacide	KAHA	⍰	✓	✓	⍰	Mild conditions
	5-oxaproline-	-Ketoacide	KAHA II	⍰	✓	✓	⍰	Introduces homoserine, epimerization
	HNSer/Thr-	-COO-salicylaldehyde	Ser/Thr ligation	✓	✓		⍰	
	H_2N-	-COOH	Lactam formation, subsequent O-to-N transfer		✓		⍰	Depsipeptide mediated
	2-OH-6-NO_2-benzyl-NH-	-COOH	Lacton formation and O-to-N migration		✓			
	H_2N- -COOH	*tert*-butyl-isocyanide and aziridine aldehyde	MCR, disrupted Ugi	✓	✓	⍰	⍰	
N-substituted amide	COOH, $-NH_2$, -CHO, -NC	-	MCR, Ugi		✓	⍰	⍰	
Amine	H_2N- nitrotyrosine	-CHO, -NC	MCR, Ugi-Smiles	✓	✓	⍰	⍰	
	$-NH_2$ (Lys)	Diborono-benzene scaffold	Petasis reachtion stapling	✓	✓	⍰	⍰	
Imine to amine	H_2N-	-CHO	Trapping imine: CN^- Strecker Reduction with BH_3CN		✓	✓		
	H_2NTrp/His-	-CHO	Trapping imine Pictet-Spengler		✓	✓		
	H_2N-	-CHO	Intramolecular 4-imidazolidinone formation		✓	✓		CyClick
	NH_2-Gly-	Carboxybenzaldehyde on sidechain	Trapping imine with dipolarophiles	✓		✓		
	NH_2 (Lys)	HCHO	Trapping with Trp or Arg		✓	✓		
Imine	H_2N-	-CHO	Trapping imine as iminoboronate		✓	✓		Introduction of non-natural amino acid; reversible upon stimuli (pH, oxidation, small molecules)
Imine to pyrrole	NH_2 (Lys)	Furan to diketoene	Ketoenal formation		✓	✓		
Oxime	hydroxylamine	aldehyde	oxime		✓	✓		E- and Z-isomer
-S-S-	Thiol	Thiol	oxidation	✓	✓	✓		
$-S-CH_2-S-$	Thiol	Thiol	Methylene thioacetal formation	⍰	✓	✓	✓	
-S-	Thiol	Bromo/chloroacetate	Attached to Lys-sidechain or N-terminal amino group	✓	✓	✓	✓	Primary amines have to be protected; can be applied on in vitro techniques
	Thiol	alkene	Thiol-ene	✓	✓	✓	✓	Alkene as acryloyl, compatible on phage

	Thiol	alkene	Radical thiol-ene	✓	✓	✓		Alkene as allyloxy PG or non-natural AA
	Thiol	alkyne	Radical thiol-yne		✓	✓		Generates E- and Z- isomer
	Thiol	DHA	Michael-addition	✓	✓	✓		
	Thiol	scaffolds		✓	✓	✓		
**C-O**	Alcohol	Aryl boronic acid	Evans-Chan-Lam coupling	⍰	✓	✓	⍰	Also for aryl amines or aryl thioethers
	Alcohol	Carbonate	Tsuji-Trost reaction	⍰	✓	✓	✓	
	Alcohol	Aryl bromide	Metal-photoredox-catalsed reactions	⍰	✓	✓	⍰	
**C-C**	Organometal, alkene	Halide	Traditional cross coupling	✓	✓	✓	⍰	Suzuki couplings on unprotected peptides reported, but usually protected
	Alkene, allyl, aryl	Aryl, aryl halide	CH activation – cross coupling	⍰	✓	✓	⍰	
	Acrylate	C-terminal carboxylic acid	Ir-photocatlysed reaction	⍰	✓	✓	✓	
	Alkyne	Alkyne	Glaser-Hay coupling	✓	✓	✓	⍰	
**C=C**	Alkene	Alkene	RCM	✓	✓	✓	✓	Also suitable for DNA-enocded libraries
**C≡C**	Alkyne	Alkyne	RCAM	✓	✓	✓	⍰	
**1,4-Disubstiuted triazole**	Alkyne	Azide	CuAAC	✓	✓	✓	✓	
**1,5-Disubstiuted triazole**	Alkyne	Azide	RuAAC	✓	✓	✓	⍰	
**1,5-Disubstiuted triazole**	Alkyne	Azide	Metal-free Dipolar cycloaddition	✓	⍰	✓	⍰	

Supplementary Table S2: Selection of examples in which peptide macorcyclizations were used to improve biological properties of peptides. AT_2 = Angiotensin II receptor type 2, AT_1 = Angiotensin II receptor type 1, OXTR = oxytocin receptor, IR = insulin receptor, pAkt = phosphorylated Akt, eIF4E = eukaryotic translation initiation factor 4E, UTR = urotensin II receptor.

Functional group or bond formed	Reaction	Aim	Change of	Target	Result	Comment	Reference
Lactam	Lactam coupling head-to-tail	β-hairpin as smaller α-helix mimetic	Secondary structure (α-helix)	p53-HDM2	IC_{50} 0.53 µM (8 AA β-hairpin) vs 1.1 µM (15 AA α-helix)		1
	Lactam sidechain-to-sidechain	Smallest α-helix (pentapeptide)			Induction of α-helix in water		2
	NCL	Improve proteolytic stability	Free N- and C-terminus	α-conotoxin MII derivatives on nicotinic acetylcholine receptor	Improved proteolytic stability (20%)	Preserve full activity (1.3 µM)	3
	Aldehyde + N-term. Cys	Synthesis natural compound		Lugdunin	S. aureus MIC 1.5 µg/mL		4
	Serine ligation	Synthesis natural compound		Daptomycin	First total synthesis		5
	Scaffold	PPI	Linear peptide	TNFα:TNFα receptor	3.1 µM (ELISA)<br>K_D 0.45 µM	scaffold essential for affinity (KD >10 µM)	6
Oxime		Affinity and proteolytic stability	Linear peptide	p53 HDM2/X	IC_{50} HDM2 110 nM; HDMX 340 nM (linear: 1.5 µM; 7.5 µM)	10- to 15- fold higher stability, 40% higher helicity	7
Disulfide	Oxidation of Cysteines		Linear vs lactam vs disulfide cyclized	ERα	K_i 0.17 µM vs 0.22 µM vs 0.025 µM	Stabilized helicity	8
Thioacetal	TBAF-mediated thioacetalization	Selectivity	Linear peptide	AT_2	selectivity over AT_1 increased from 0.5-fold to 10-fold	Change of affinity strongly dependent on linker length;	9
	CH_2I_2-mediated thioacetalization		Disulfide	OXTR	6-fold decrease in affinity	only weak decrease in affinity, serum stability increased 2.5-fold	10
	CH_2I_2-mediated thioacetalization	Affinity and serum stability	Disulfide	IR	2-fold decrease in pAkt signalling, but strongly increased serum stability		11
Thioether	Scaffold	Activity, helicity	Linear vs stapled peptide	Mdm2/Mdmx	Mdm2: IC_{50} 57 nM vs 5.4 nM<br>Mdmx: IC_{50} 1800 nM vs 14 nM	Increased cell permeability of stapled peptides	12
	Photo controllable scaffold	Activity, helicity	Linear vs trans vs cis	Bcl-xl	K_D 134 nM vs 825 nM vs 42 nM	Opportunity to activate apoptotic process under light control	13
C-C single bond	$C(sp^3)$–H activation-mediated macrocyclization	Binding, stability	Linear peptide	Integrins	>100-fold increased proteolytic stability	When applied to RGD-containing peptides: binding to αvβ3 integrin-overexpressing cells strongly increased	14)
Alkene	RCM	Affinity	Linear peptide	eIF4E	6-fold increase in affinity	Affinity dependent on ring size	15
	RCM	Activity, stability	Linear peptide	Bcl-xl	K_D 154 nM vs 69 nM; stability 60 fold improved	intramolecular H-bond mimetic	16
	RCM	Affinity, helicity	Linear peptide	p53 MDM2/X	K_i (MDM2) 14 nM vs 1 nM,<br>K_i (MDX) 47 nM vs 7 nM		17

		Insulin Activity	Disulfide	IR	Cis-Isomer showed increased efficacy in mice compared to native insulin	Binding of trans-isomer to IR (A and B) 50-fold reduced	[18]
Alkyne	RCAM	Affinity	Linear peptide	Rab8a	>10-fold increase in affinity	Affinity strongly dependent on linker lenght	[19]
1,4-substituted 1,2,3-triazole	CuAAC	Affinity, helicity	Linear peptide	BCL9 β-catenin	K_i 0.6 µM vs 0.13 µM; Helicity 44% vs 90%	improved stability	[20]
	CuAAC	Activity	Disulfide		Equipotent as the disulfide in rat model for neuropathic pain	Drastically increased plasma stability	[21]
1,5-substituted 1,2,3-triazole	RuAAC	Affinity	Disulfide	UTR	Maintained affinity	1,4-substituted triazole analogues lost affinity	[22]
	RuAAC	Inhibitory activity, hepatic stability	Disulfide	Set of proteases	Reduced inhibition 5-100-fold compared to disulfide; hepatic stability of triazoles increased >2 - >6-fold		[23]

Supplementary Table S3: Example reaction conditions used for in solution RCM for peptides. stochi. = stochiometric, TCE = 1,1,2-trichloroethane, TFE = 1,1,1-trifluoroethanol, rt = room temperature

Substrate conc.	Catalyst	Catalyst loading	Solvent	T (°C)	other conditions	t	Yields	workup	Comment	Ref.
5 mM	Grubbs I	20%	$CHCl_3$	25 °C		3 -4 h	85-90%			[24]
5 mM	Hoveyda-Grubbs I	2.50%	DCM	40 °C		20 h	85%			[25]
10 mM	Hoveyda-Grubbs I	5%	DCM	40 °C (reflux)		24 h	87%			[26]
2.5 mM	Grubbs II	stochi.	TCE/DMF				67%			[27]
7 mM	Grubbs I	20%	DCM	rt	dry, degassed	48 h	40%			[28]
0.6 mM	Grubbs II	10%	DCM	reflux	dry	24 h	9% (overall: SPPS, cleavage, macrolactamizstion, RCM)	Removed solvent	Gave specifically cis alkene	[29]
4 mM	Grubbs II	15-20%	DCM	rt		6-7.5 h	42-52%		Catalyst added in two portions, second portion after 2- 3 h	[30]
10 mM	Hoveyda-Grubbs II	20%	TFE/DCM (4:1)	**rt**		48 h	20-30%	Removed solvent, purified by HPLC		[31]

Supplementary Table S4: Example reaction conditions used for on resin RCM for peptides. † = nominal concentration (resin loading/solvent volume), DCE = 1,2-dichloroethane, TCE = 1,1,2-trichloroethane, rt = room temperature, µW = microwave, * = not specified which generation.

Substrate conc.†	Catalyst	Catalyst loading	Additives (eq)	Solvent	T (°C)	Other conditions	t	Yields	Workup	Comment	Ref.
	Grubbs II	15%		DCM	100 °C		75 min				32
82 mM	Hoveyda-Grubbs II	20%		DCM	100 °C	µW (300 W)	1 h	29%		Was essential to change from chlorotritiyl to HMBA resin	33
	Hoveyda-Grubbs II	15%		DCM/DMF	100 °C	µW (120 W)	2 h	7 - 16%			34
5.5 mM	Grubbs II	18%	2,6-Dichlorotoluene (3.6 eq)	TCE	reflux	N_2	16 h	45		Mixture of E/Z (9%/37%)	35
33.3 mM	Hoveyda-Grubbs*	200 g/mol (x2)		TCE	50 °C		overnight				36
13 mM	Grubbs I	40%		DCM	rt	Ar	48 h				37
24 mM	Grubbs II	20%	LiCl (0.8 eq.)	DCM/DMF (95/5)	100 °C	µW (100 W)	2 h	95%		RCM was unsuccesful on full length peptide, needed to be done on truncated version which was afterwards elongated, 3:1 E:Z	38
33.3 mM	Grubbs II	10%	LiCl (4 eq.)	DCM:DMF (4:1)	40 °C, reflux	dry, degassed	48 h	22% (overall: SPPS, RCM, cleavage)	Washed with DMSO for 12 h after RCM, filtration, washed with DCM, MeOH		29
	Hoveyda-Grubbs II			DCE	50 °C		8 h		Washed with DMF, DCM		17
	Grubbs II	20%	LiCl	DCM	100 °C	µW (60 W)	1 h				39
24 mM	Grubbs II	20%	LiCl (0.8)	DCM:DMF (19:1 or 20:1)	100 °C	µW (80 - 100 W)	1 - 2 h	100%			40
	Hoveyda-Grubbs II	30%		DCM:DMF (4:1)	100 °C	µW (100 W)	2 h	26% (crude)			41
	Hoveyda-Grubbs II	20-35%		TFE:DCM (4:1)	rt		48 h	20-30% (overall, convsersion > 90%)	Wash with DMSO:DMF (1:1) overnight	Reaction on Novapeg resin required only 20% catalyst, on Wang resin 35%	31

Supplementary Table S5: Example reaction conditions used for solution CuAAC for peptides. TFE = 1,1,1-trifluoroethanol, TBTA = tris[(1-benzyl-1*H*-1,2,3-triazol-4-yl)methyl]amine, THTPA = tris(3-hydroxypropyltriazolylmethyl)amine, rt = room temperature.

Substrate conc.	Cu salt (eq)	Ascorbate (eq)	Additives (eq)	Solvent	T	Other conditions	time	Yield	Workup	Comment	Ref.
1 mM	$CuSO_4$ x 5 H_2O (3)	6		NH_4CO_3 buffer (0.1 M)	rt	Ar	30 min		pH 2 (TFA), SPE (C18)		42
1.2 M (!)	CuBr (1)	-	DBU (3)	DCM	rt	dry, N_2	12 h		Washed with 3 M HCl, extracted with DCM		43
0.775 mM	$CuSO_4$ x 5 H_2O (14)	Large excess		H_2O:*t*BuOH (2:1)	rt		Overnight		Concentrated, lyophilise, C18 HPLC		44
0.5 mM	$CuSO_4$ x 5 H_2O (14)	13		H_2O:*t*BuOH (2:1)	rt		1 h		HPLC (C12)		45
0.954 mM	$CuSO_4$ x 5 H_2O (8.4)	8.45		H_2O:*t*BuOH (2:1)	rt		overnight		Concentrated, lyophilise, C18 HPLC (SPE)		46
1 mM	CuBr (0.2)	-	DBU (3)	$PhCH_3$	110 °C		16 h	70%		CuI gave substantial iodoatriazole adducts	47
0.15 mM	CuI.P(OEt)$_3$ (0.31)	-	DIPEA (3)	DCM	rt	Protected from light	42 h	83%			48
0.3 mM	$CuSO_4$ x 5 H_2O (4.4)	4.4	-	H_2O:*t*BuOH (2:1)	rt		Overnight				49
1 mM	Cu(MeCN)$_4$][PF$_6$] (1.8)	2.1	Tris-tri(methylazolyl)-amine (2.7)	10 mM Na-Phosphate, pH 8	rt	Protected from light	Overnight				50
10 mM	Cu(MeCN)$_4$][PF$_6$] (0.05)	-	TBTA (0.05)	DCM	55 °C		12 h	39-79%			51
0.8 mM	$CuSO_4$ x 5 H_2O (4)	6	-	H_2O:*t*BuOH (1:1)	rt		Overnight				36
10 mg/mL	CuBr (0.01)	-	DBU (0.03)	DCM	rt		6 h				52
1 mM	CuI (2)	-	DIPEA (2), 2,6-lutidine (2)	MeCN	rt	Ar, degassed	12 h				53
0.5 mM	$CuSO_4$ (1)	5	THTPA (1)	H_2O:*t*BuOH (1:1) or H_2O:TFE (1:1)	rt		< 1 h	13-51%			54
1 mg/mL	$CuSO_4$ (1)	1	DIPEA (8)	H_2O	rt	Degassed	12 h				22
0.1 mM	Cu(MeCN)$_4$][PF$_6$] (0.95)	-		THF:MeOH (1:1)	40 °C	Degassed	20 h	64-83%			55
0.5 mM	$CuSO_4$ (2)	10	THTPA (2)	DMF/H_2O (2:1)	rt		5 min				56,57
0.8 mg/mL	$CuSO_4$ x 5 H_2O (1)	3	THTPA (1)	H_2O:*t*BuOH (1:1)	rt	N_2, degassed		52-92%	Diluted with H_2O, lyophilised, HPLC		58
1.5 mM	$CuSO_4$ (10)	10	TBTA (10)	H_2O:*t*BuOH (1:1)	rt		12 h		HPLC		59
0.91 mg/mL	$CuSO_4$ x 5 H_2O (1)	3	THTPA (1)	H_2O:*t*BuOH (1:1)	rt	N_2	16 h				60
1 mg/mL	$CuSO_4$ x 5 H_2O (1)	3	THTPA (1)	H_2O:*t*BuOH (1:1)	rt	N_2	16 h				61
	$CuSO_4$ x 5 H_2O (1)	3	THTPA (1)	H_2O:*t*BuOH (1:1)	rt	N_2	15 min - 2 h				62

	$CuSO_4$ x 5 H_2O (1)	3	THTPA (1)	H_2O:*t*BuOH (1:1)	rt	N_2, degassed	16 h				[63]
0.01 mM	$CuSO_4$ (4)	4	-	buffer pH 8 (250 mM Na borate, 1M NaCl)	60 °C		30 min				[64]
1 mM	CuBr (0.8)	-	-	$PhCH_3$	Reflux	Ar	Overnight	43%	Celite filtration, DCM wash of filter, flash column chromatography		[65].
1.1 mM	CuI.P(OEt)$_3$ (1.5)	-	-	DCM	rt	Protected from light	5 days	75%	Solvent removal, flash chromatography		[66]
1 mM	Cu(MeCN)$_4$][PF$_6$] (1.5)	-		$PhCH_3$/MeOH (4:1)	rt	N_2	24 h	0-46%	Solvent removal, flash chromatography	Yield depended on chain length of employed azide	[67]
0.35 mM	$CuSO_4$ (2)	10	THTPA (10)	H_2O	37 °C	N_2	3 h	45-55%	Monitored by HPLC, purification by HPLC		[23]
1 mg/mL	$CuSO_4$ x 5 H_2O (1)	1	DIPEA (8)	H_2O	rt	Ar, degassed	Overnight	7.5-12.8% (overall)	Lyophilisation, HPLC prufication by HPLC		[68]
	$CuSO_4$ x 5 H_2O (2)	3	THTPA (1) for some	H_2O:*t*BuOH (1:1)	rt	N_2 ,degassed	overnight		SPE, HPLC purification	.	[69]
2 mM	$CuSO_4$ x 10 H_2O (2.5)	10		H_2O:*t*BuOH (1:1.5)			overnight		Removal tBuOH, filtration, HPLC purifcation		[70].
1 mM	CuBr (0.2)	-	DBU (3)	$PhCH_3$	reflux	Ar, degassed	16 h	36-56%	Celite filtration, DCM wash of filter, flash column chromatography	Head-to-tail, tetrapeptide	[71]
0.2 mM	CuI (2)	-	TBTA (2), DIPEA (2), 2,6-lutidine (2)	MeCN	rt	Ar, degassed	48 h		Removed solvent, HPLC purification	Head-to-tail, tetrapeptide	[72]
1 mM	CuI (0.33)	-	DIPEA (3), 2,6-lutidine (2)	MeCN:THF (4:1)	rt	Degassed	14 h	57-92%	Filtration, removal of solvent, HPLC purification	Head-to-tail, pentapeptide	[73]
	CuBr (1)	1	DIPEA (10), 2,6-lutidine (10)	DMF:H_2O	rt		18 h		Washed with DMF, DCM		[74]

Supplementary Table S6: Example reaction conditions used for on-resin CuAAC for peptides. TBTA = tris[(1-benzyl-1*H*-1,2,3-triazol-4-yl)methyl]amine, † = nominal concentration (resin loading/solvent volume), rt = room temperature.

Substrate conc.†	Cu salt (eq)	Ascorbate (eq	Additives (eq)	Solvent	T	Other conditions	Time	Yield	Workup	Ref.
15 µL/mg resin	CuI (2)	-	DIPEA (50 eq)	THF			16 h	76%		75
625 mL/mmol	CuI (0.5)	1	2,6-Lutidine (2 eq)	NMP/H_2O (4:1)			48 - 96 h	8.4%		76
20 mg/mL	CuBr (1)	1	2,6-Lutidine (10 eq), DIPEA (10 eq)	DMSO	rt	Degassed	16 h			77
	CuBr	yes	2,6-Lutidine, DIPEA	DMF/MeCN	rt		8 h	22% (overall)		78
	CuBr (1)	3	2,6-Lutidine (10 eq), DIPEA (10 eq)	DMF/MeCN (10/3)	rt	Degassed	6 h	20%-75%		79
	CuI (2)	2	DIPEA (3)	DMF	rt		Overnigh t			36
	$[Cu(CH_3CN)_4]$ $[PF_6]$ (1)	-	TBTA (1), DIPEA (2)	DMF			24 h			80
	CuI (1.5)	7 (as ascorbic acid)	Piperidine (20 vol%)	DMF	rt	Light protected	15 h		Wash with 5% sodium diethyldithiocarbaamte, 5% DIEPA in DMF, MeOH, DMF, NMP	81

Supplementary Table S7: Example reaction conditions used for in solution RuAAC for peptides. cod = 1,5-cycloocatadien, rt = room temperature.

Substrate conc.	Catalyst	Catalyst loading	Solvent	T	Other conditions	Time	Yield	Workup	Comment	Ref.
1.9 mM	$[Cp^*RuCl]_4$	3.75%	DMF	115 °C	µW (200 W)	30 min	10%	Removal of DMF in vacuo, extraction DCM/water, silica column chromatography followed by RP-8	Desired product only minor, major product correct m/z, but no symmetry in NMR --> probably 1,4-analogue	82
5 mM	$[Cp^*RuCl]_4$	30%	THF/MeOH	50 °C	-	24 h	40%			83
5 mM	$[Cp^*RuCl]_4$	15%	THF/MeOH (95:5)	50 °C	N_2	24 h	14-68%	Removal of solvent, flash chromatography		84
3.1 mM	Cp*RuCl(cod)	50%	DMF	80 °C	dry	18 h	3.4-7.0% (overall: SPPS, RuAAC, deprotection)	Addition of DCM, washed organic phase with H_2O+0.05% TFA, removal of solvent, redissolved in H_2O/MeCN (1:1)+0.05% TFA, lyophilised	Sidechain protected peptide	85

Supplementary Table S8: Example reaction conditions used for on-resin solution RuAAC for peptides. † = nominal concentration (resin loading/solvent volume), cod = 1,5-cycloocatadien

Substrate conc.†	Catalyst	Catalyst loading	Solvent	T	Other conditions	Time	Yield	Workup	Ref.
200 mM	Cp*RuCl(cod)	3.75%	DMF	60 °C	Dry, degassed, Ar	6 h		Wash with DMF, MeOH, DCM	22
< 12.5 mM	Cp*RuCl(cod)	30%	DMF	60 °C	µW (30 W)	5 h	2.1% (overall)	Wash with MeOH, 0.5% dietyhldithiocarbamate in DMF, DMF, DCM	68
25 mM	Cp*RuCl(cod)	15%	DMF	70 °C	Dry, degassed	1 h	8-21%	Monitored by IR, continued with Fmoc-deprotection	86
	Cp*RuCl(cod)	50%	DMF	60 °C	µW (30 W), N_2	3 h		Wash with MeOH, 2% dietyhldithiocarbamate in DMF, DMF	69
75 mM	Cp*RuCl(PPh_3)$_2$		DMF	65 °C	µW, degassed, Ar	2.5 h		Wash with DMF, Et_2O	70

1 R. Fasan, R. L. A. Dias, K. Moehle, O. Zerbe, J. W. Vrijbloed, D. Obrecht and J. A. Robinson, *Angew. Chemie - Int. Ed.*, 2004, **43**, 2109–2112.
2 N. E. Shepherd, H. N. Hoang, G. Abbenante and D. P. Fairlie, *J. Am. Chem. Soc.*, 2005, **127**, 2974–2983.
3 R. J. Clark, H. Fischer, L. Dempster, N. L. Daly, K. J. Rosengren, S. T. Nevin, F. A. Meunier, D. J. Adams and D. J. Craik, *Proc. Natl. Acad. Sci. U. S. A.*, 2005, **102**, 13767–13772.
4 A. Zipperer, M. C. Konnerth, C. Laux, A. Berscheid, D. Janek, C. Weidenmaier, M. Burian, N. A. Schilling, C. Slavetinsky, M. Marschal, M. Willmann, H. Kalbacher, B. Schittek, H. Brötz-Oesterhelt, S. Grond, A. Peschel and B. Krismer, *Nature*, 2016, **535**, 511–516.
5 H. Y. Lam, Y. Zhang, H. Liu, J. Xu, C. T. T. Wong, C. Xu and X. Li, *J. Am. Chem. Soc.*, 2013, **135**, 6272–6279.
6 W. Lian, P. Upadhyaya, C. A. Rhodes, Y. Liu and D. Pei, *J. Am. Chem. Soc.*, 2013, **135**, 11990–11995.
7 J. M. Smith, J. R. Frost and R. Fasan, *Chem. Commun.*, 2014, **50**, 5027–5030.
8 A. M. Leduc, J. O. Trent, J. L. Wittliff, K. S. Bramlett, S. L. Briggs, N. Y. Chirgadze, Y. Wang, T. P. Burris and A. F. Spatola, *Proc. Natl. Acad. Sci. U. S. A.*, 2003, **100**, 11273–11278.
9 S. Lindman, G. Lindeberg, P. A. Frändberg, F. Nyberg, A. Karlén and A. Hallberg, *Bioorganic Med. Chem.*, 2003, **11**, 2947–2954.
10 C. M. B. K. Kourra and N. Cramer, *Chem Sci*, 2016, **7**, 7007–7012.
11 N. Zheng, P. Karra, M. A. Vandenberg, J. H. Kim, M. J. Webber, W. L. Holland and D. H.-C. Chou, *J. Med. Chem.*, 2019, **62**, 11437–11443.
12 A. Muppidi, Z. Wang, X. Li, J. Chen and Q. Lin, *Chem. Commun.*, 2011, **47**, 9396–9398.
13 S. Kneissl, E. J. Loveridge, C. Williams, M. P. Crump and R. K. Allemann, *Chembiochem*, 2008, **9**, 3046–3054.
14 J. Tang, Y. He, H. Chen, W. Sheng and H. Wang, *Chem. Sci.*, 2017, **8**, 4565–4570.
15 J. M. Song, E. E. Gallagher, A. Menon, L. D. Mishra and A. L. Garner, *Org. Biomol. Chem.*, 2019, **17**, 6414–6419.
16 D. Wang, W. Liao and P. S. Arora, *Angew. Chemie - Int. Ed.*, 2005, **44**, 6525–6529.
17 Y. S. Chang, B. Graves, V. Guerlavais, C. Tovar, K. Packman, K. H. To, K. A. Olson, K. Kesavan, P. Gangurde, A. Mukherjee, T. Baker, K. Darlak, C. Elkin, Z. Filipovic, F. Z. Qureshi, H. Cai, P. Berry, E. Feyfant, X. E. Shi, J. Horstick, D. A. Annis, A. M. Manning, N. Fotouhi, H. Nash, L. T. Vassilev and T. K. Sawyer, *Proc. Natl. Acad. Sci. U. S. A.*, 2013, **110**, E3445–E3454.
18 B. Van Lierop, S. Chee Ong, A. Belgi, C. Delaine, S. Andrikopoulos, N. L. Haworth, J. G. Menting, M. C. Lawrence, A. J. Robinson and B. E. Forbes, *Sci. Rep.*, 2017, **7**, 1–13.
19 P. M. Cromm, S. Schaubach, J. Spiegel, A. Fürstner, T. N. Grossmann and H. Waldmann, *Nat. Commun.*, 2016, **7**, 11300.
20 S. A. Kawamoto, A. Coleska, X. Ran, H. Yi, C. Y. Yang and S. Wang, *J. Med. Chem.*, 2012, **55**, 1137–1146.
21 A. Gori, C. I. A. Wang, P. J. Harvey, K. J. Rosengren, R. F. Bhola, M. L. Gelmi, R. Longhi, M. J. Christie, R. J. Lewis, P. F. Alewood and A. Brust, *Angew. Chemie - Int. Ed.*, 2015, **54**, 1361–1364.
22 S. Pacifico, A. Kerckhoffs, A. J. Fallow, R. E. Foreman, R. Guerrini, J. Mcdonald, D. G. Lambert and A. G. Jamieson, *Org. Biomol. Chem*, 2017, **15**, 4704–4710.
23 A. M. White, S. J. de Veer, G. Wu, P. J. Harvey, K. Yap, G. J. King, J. E. Swedberg, C. K. Wang, R. H. P. Law, T. Durek and D. J. Craik, *Angew. Chemie - Int. Ed.*, 2020, **59**, 11273–11277.
24 H. E. Blackwell and R. H. Grubbs, *Angew. Chemie Int. Ed.*, 1998, **37**, 3281–3284.
25 M. Poirier, N. Aubry, C. Boucher, J.-M. Ferland, S. Laplante and Y. S. Tsantrizos, *J. Org. Chem.*, 2005, **70**, 10765–10773.
26 N. K. Yee, V. Farina, I. N. Houpis, N. Haddad, R. P. Frutos, F. Gallou, X.-J. Wang, X. Wei, R. D. Simpson, X. Feng, V. Fuchs, Y. Xu, J. Tan, L. Zhang, J. Xu, L. L. Smith-Keenan, J. Vitous, M. D.

Ridges, E. M. Spinelli, M. Johnson, K. Donsbach, T. Nicola, M. Brenner, E. Winter, P. Kreye and W. Samstag, *J. Org. Chem.*, 2006, **71**, 7133–7145.
27 H. T. Ten Brink, D. T. S. Rijkers and R. M. J. Liskamp, *J. Org. Chem.*, 2006, **71**, 1817–1824.
28 M. N. Islam, M. S. Islam, M. A. Hoque, T. Kato, N. Nishino, A. Ito and M. Yoshida, *Bioorganic Med. Chem.*, 2014, **22**, 3862–3870.
29 S. Jiang, P. Li, S.-L. Lee, C. Y. Lin, Y.-Q. Long, M. D. Johnson, R. B. Dickson and P. P. Roller, *Org. Lett.*, 2007, **9**, 9–12.
30 Ø. Jacobsen, J. Klaveness, O. Petter Ottersen, M. Reza Amiry-Moghaddam and P. Rongved, *Org. Biomol. Chem.*, 2009, **7**, 1599–1611.
31 Y. E. Bergman, M. P. Del Borgo, R. D. Gopalan, S. Jalal, S. E. Unabia, M. Ciampini, D. J. Clayton, J. M. Fletcher, R. J. Mulder, J. A. Wilce, M. I. Aguilar and P. Perlmutter, *Org. Lett.*, 2009, **11**, 4438–4440.
32 M. P. C. Mulder, J. A. W. Kruijtzer, E. J. Breukink, J. Kemmink, R. J. Pieters and R. M. J. Liskamp, *Bioorganic Med. Chem.*, 2011, **19**, 6505–6517.
33 P. Martín-Gago, R. Ramón, E. Aragón, J. Fernández-Carneado, P. Martin-Malpartida, X. Verdaguer, P. López-Ruiz, B. Colás, M. A. Cortes, B. Ponsati, M. J. Macias and A. Riera, *Bioorganic Med. Chem. Lett.*, 2014, **24**, 103–107.
34 Z. Amso, R. Kowalczyk, Y.-E. Park, M. Watson, J.-M. Lin, D. S. Musson, J. Cornish and M. A. Brimble, *Org. Biomol. Chem.*, 2016, **14**, 6231–6243.
35 F. J. Dekker, N. J. De Mol, M. J. E. Fischer, J. Kemmink and R. M. J. Liskamp, *Org. Biomol. Chem*, 2003, **1**, 3297–3303.
36 L. Frankiewicz, C. Betti, K. Guillemyn, D. Tourwé, Y. Jacquot and S. Ballet, *J. Pept. Sci.*, 2013, **19**, 423–432.
37 R. J. Platt, T. S. Han, B. R. Green, M. D. Smith, J. Skalicky, P. Gruszczyński, H. S. White, B. Olivera, G. Bulaj and J. Gajewiak, *J. Biol. Chem.*, 2012, **287**, 20727–20736.
38 B. Van Lierop, S. Chee Ong, A. Belgi, C. Delaine, S. Andrikopoulos, N. L. Haworth, J. G. Menting, M. C. Lawrence, A. J. Robinson and B. E. Forbes, *Sci. Rep.*, 2017, **7**, 17239.
39 C. A. MacRaild, J. Illesinghe, B. J. Van Lierop, A. L. Townsend, M. Chebib, B. G. Livett, A. J. Robinson and R. S. Norton, *J. Med. Chem.*, 2009, **52**, 755–762.
40 S. Chhabra, A. Belgi, P. Bartels, B. J. van Lierop, S. D. Robinson, S. N. Kompella, A. Hung, B. P. Callaghan, D. J. Adams, A. J. Robinson and R. S. Norton, *J. Med. Chem.*, 2014, **57**, 9933–9944.
41 R. Kowalczyk, S. H. Yang, M. A. Brimble, K. E. Callon, M. Watson, Y. E. Park and J. Cornish, *Bioorganic Med. Chem.*, 2014, **22**, 3565–3572.
42 M. Horn, F. Reichart, S. Natividad-Tietz, D. Diaz and I. Neundorf, *Chem. Commun.*, 2016, **52**, 2261–2264.
43 P. M. Weerawarna, Y. Kim, A. C. Galasiti Kankanamalage, V. C. Damalanka, G. H. Lushington, K. R. Alliston, N. Mehzabeen, K. P. Battaile, S. Lovell, K.-O. Chang and W. C. Groutas, *Eur. J. Med. Chem.*, 2016, **119**, 300–318.
44 A. L. C. Isaad, A. M. Papini, M. Chorev and P. Rovero, *J. Pept. Sci.*, 2009, **15**, 451–454.
45 V. Celentano, D. Diana, C. Di Salvo, L. De Rosa, A. Romanelli, R. Fattorusso and L. D. D'Andrea, *Chem. – A Eur. J.*, 2016, **22**, 5534–5537.
46 C. Testa, M. Scrima, M. Grimaldi, A. M. D'Ursi, M. L. Dirain, N. Lubin-Germain, A. Singh, C. Haskell-Luevano, M. Chorev, P. Rovero and A. M. Papini, *J. Med. Chem.*, 2014, **57**, 9424–9434.
47 V. D. Bock, R. Perciaccante, T. P. Jansen, H. Hiemstra and J. H. Van Maarseveen, *Org. Lett.*, 2006, **8**, 919–922.
48 Ø. Jacobsen, H. Maekawa, N.-H. Ge, C. H. G€ Orbitz, P. P. Rongved, O. P. Ottersen, M. Amiry-Moghaddam and J. Klaveness, *J. Org. Chem*, 2011, **76**, 1228–1238.
49 S. Cantel, A. Le, C. Isaad, M. Scrima, J. J. Levy, R. D. Dimarchi, P. Rovero, J. A. Halperin, A. Maria D'ursi, A. M. Papini and M. Chorev, *J. Org. Chem*, 2008, **73**, 5663–5674.
50 J. H. Park and M. L. Waters, *Org. Biomol. Chem.*, 2013, **11**, 69–77.
51 G. Chouhan and K. James, *Org. Lett.*, 2013, **15**, 1206–1209.
52 Y. Liu, L. Zhang, J. Wan, Y. Li, Y. Xu and Y. Pan, *Tetrahedron*, 2008, **64**, 10728–10734.
53 J. H. Van Maarseveen, W. S. Horne and M. R. Ghadiri, *Org. Lett.*, 2005, **7**, 4503–4506.
54 P. T. Tran, C. Ø. Larsen, T. Røndbjerg, M. De Foresta, M. B. A. Kunze, A. Marek, J. H. Løper, L.-E. Boyhus, A. Knuhtsen, K. Lindorff-Larsen and D. S. Pedersen, *Chem. - A Eur. J.*, 2017, **23**, 3490–3495.
55 M. Strack, É. Billard, D. Chatenet and W. D. Lubell, *Bioorganic Med. Chem. Lett.*, 2017, **27**, 3412–3416.
56 G. J. J. Richelle, S. Ori, H. Hiemstra, J. H. van Maarseveen and P. Timmerman, *Angew. Chemie - Int. Ed.*, 2018, **57**, 501–505.
57 G. J. J. Richelle, M. Schmidt, H. Ippel, T. M. Hackeng, J. H. van Maarseveen, T. Nuijens and P. Timmerman, *ChemBioChem*, 2018, **19**, 1934–1938.
58 J. Iegre, P. Brear, D. J. Baker, Y. S. Tan, E. L. Atkinson, H. F. Sore, D. H. O' Donovan, C. S. Verma, M. Hyvönen, H. Hyvönen and D. R. Spring, *Chem. Sci.*, 2019, **10**, 5056–5063.
59 L. Zhang, T. Navaratna, J. Liao and G. M. Thurber, *Bioconjug. Chem.*, 2015, **26**, 329–337.
60 M. M. Wiedmann, Y. S. Tan, Y. Wu, S. Aibara, W. Xu, H. F. Sore, C. S. Verma, L. Itzhaki, M. Stewart, J. D. Brenton and D. R. Spring, *Angew. Chemie - Int. Ed.*, 2017, **56**, 524–529.

61 Y. H. Lau, P. De Andrade, S.-T. Quah, M. Rossmann, L. Laraia, N. Sköld, S. Sköld, T. J. Sum, P. J. E. Rowling, T. L. Joseph, C. Verma, M. Hyvönen, H. Hyvönen, L. S. Itzhaki, A. R. Venkitaraman, C. J. Brown, D. P. Lane and D. R. Spring, *Chem. Sci.*, 2014, **5**, 1804–1809.
62 J. Iegre, N. S. Ahmed, J. S. Gaynord, Y. Wu, K. M. Herlihy, Y. S. Tan, M. E. Lopes-Pires, R. Jha, Y. H. Lau, H. F. Sore, C. Verma, D. H. O' Donovan, N. Pugh and D. R. Spring, *Chem. Sci.*, 2018, **9**, 4638–4643.
63 W. Xu, Y. H. Lau, G. Fischer, Y. S. Tan, A. Chattopadhyay, M. De La Roche, M. Hyvö, C. Verma, D. R. Spring and L. S. Itzhaki, *J. Am. Chem. Soc.*, 2017, **139**, 2245–2256.
64 Y. Onda, G. Bassi, A. Elsayed, F. Ulrich, S. Oehler, L. Plais, J. Scheuermann and D. Neri, *Chem. – A Eur. J.*, , DOI:10.1002/chem.202005423.
65 Q. Luo, Y. Tao, W. Sheng, J. Lu and H. Wang, *Nat. Commun.*, 2019, **10**, 1–9.
66 F. Tahoori, S. Balalaie, R. Sheikhnejad, M. Sadjadi and P. Boloori, *Amino Acids*, 2014, **46**, 1033–1046.
67 J. Zhang, J. Kemmink, D. T. S. Rijkers and R. M. J. Liskamp, *Org. Lett.*, 2011, **13**, 3438–3441.
68 M. Empting, O. Avrutina, R. Meusinger, S. Fabritz, M. Reinwarth, M. Biesalski, S. Voigt, G. Buntkowsky and H. Kolmar, *Angew. Chemie - Int. Ed.*, 2011, **50**, 5207–5211.
69 S. R. Tala, A. Singh, C. J. Lensing, S. M. Schnell, K. T. Freeman, J. R. Rocca and C. Haskell-Luevano, *ACS Chem. Neurosci.*, 2017, **9**, 1001–1013.
70 S. Tomassi, A. M. Trotta, C. Ieranò, F. Merlino, A. Messere, G. Rea, F. Santoro, D. Brancaccio, A. Carotenuto, V. M. D'Amore, F. S. Di Leva, E. Novellino, S. Cosconati, L. Marinelli, S. Scala and S. Di Maro, *Chem. - A Eur. J.*, 2020, **26**, 10113–10125.
71 V. D. Bock, D. Speijer, H. Hiemstra and J. H. Van Maarseveen, *Org. Biomol. Chem.*, 2007, **5**, 971–975.
72 W. S. Horne, C. A. Olsen, J. M. Beierle, A. Montero and M. R. Ghadiri, *Angew. Chemie - Int. Ed.*, 2009, **48**, 4718–4724.
73 T. S. Hu, R. Tannert, H. D. Arndt and H. Waldmann, *Chem. Commun.*, 2007, 3942–3944.
74 C. Testa, D. D'addona, M. Scrima, A. M. Tedeschi, A. M. D'ursi, C. Bernhard, F. Denat, C. Bello, P. Rovero, M. Chorev and A. M. Papini, *Pept. Sci.*, , DOI:10.1002/pep2.24071.
75 M. Roice, I. Johannsen and M. Meldal, *QSAR Comb. Sci.*, 2004, **23**, 662–673.
76 V. Goncalves, B. Gautier, A. Regazzetti, P. Coric, S. Bouaziz, C. Garbay, M. Vidal and N. Inguimbert, *Bioorg. Med. Chem. Lett.*, 2007, **17**, 5590–5594.
77 S. Ingale and P. E. Dawson, *Org. Lett.*, 2011, **13**, 2822–2825.
78 B. B. Metaferia, M. Rittler, J. S. Gheeya, A. Lee, H. Hempel, A. Plaza, W. G. Stetler-Stevenson, C. A. Bewley and J. Khan, *Bioorganic Med. Chem. Lett.*, 2010, **20**, 7337–7340.
79 R. A. Turner, A. G. Oliver and R. S. Lokey, *Org. Lett.*, 2007, **9**, 5011–5014.
80 C. Ngambenjawong, J. M. B. Pineda and S. H. Pun, *Bioconjug. Chem.*, 2016, **27**, 2854–2862.
81 R. Kandler, S. Das and A. Nag, *RSC Adv.*, 2021, **11**, 4842–4852.
82 M. R. Krause, R. Goddard and S. Kubik, *J. Org. Chem.*, 2011, **76**, 7084–7095.
83 Y. Zhang, C. Xu, H. Y. Lam, C. L. Lee and X. Li, *Proc. Natl. Acad. Sci. U. S. A.*, 2013, **110**, 6657–6662.
84 W. Wu, Z. Zhang and L. S. Liebeskind, *J. Am. Chem. Soc.*, 2011, **133**, 14256–14259.
85 C. J. White and A. K. Yudin, *Nat. Chem.*, 2011, **3**, 509–524.
86 A. Knuhtsen, C. Whitmore, F. S. Mcwhinnie, L. Mcdougall, R. Whiting, B. O. Smith, C. M. Timperley, A. C. Green, K. I. Kinnear and A. G. Jamieson, *Chem. Sci.*, 2019, **10**, 1671–1676.
